# Cobalt Single‐Atom Intercalation in Molybdenum Disulfide Enhances Piezocatalytic and Enzyodynamic Activities for Advanced Cancer Therapeutics

**DOI:** 10.1002/advs.202415485

**Published:** 2025-02-14

**Authors:** Haomiao Bai, Sujun Ding, Yanfei Dai, Jiefu Liu, Huangjing Chen, Wei Feng, Dehong Yu, Yu Chen, Xuejun Ni

**Affiliations:** ^1^ Department of Medical Ultrasound Affiliated Hospital of Nantong University Nantong 226001 P. R. China; ^2^ Radiology Department Branch of Affiliated Hospital of Nantong University Nantong 226001 P. R. China; ^3^ Materdicine Lab School of Life Sciences Shanghai University Shanghai 200444 P. R. China; ^4^ Shanghai Institute of Materdicine Shanghai 200051 P. R. China

**Keywords:** enzyodynamic therapy, nanomedicine, piezocatalytic therapy, single‐atom doping

## Abstract

Piezoelectric semiconductor nanomaterials have attracted considerable interest in piezocatalytic tumor treatment. However, piezocatalytic therapy encounters obstacles such as suboptimal piezoelectric responses, rapid electron‐hole recombination, inefficient energy harvesting, and the complexities of the tumor microenvironment. In this study, sulfur vacancy‐engineered cobalt (Co) single‐atom doped molybdenum disulfide (SA‐Co@MoS_2_) nanoflowers are strategically designed, which exhibit enhanced piezoelectric effects. Specifically, the introduction of Co single atom not only induces lattice distortion and out‐of‐plane polarization but also leads to the formation of numerous sulfur vacancies. These changes collectively narrow the intrinsic bandgap of the material, facilitating effective separation and migration of charge carriers, and enabling efficient production of reactive oxygen species under ultrasound stimulation. Additionally, the SA‐Co@MoS_2_ nanoflowers demonstrate improved enzymatic activity and exhibit glutathione depletion capabilities attributed to the mixed valence states of Co, intensifying oxidative stress in tumor cells, and leading to cell cycle arrest and apoptosis, while the inactivation of glutathione peroxidase 4 induces ferroptosis. Both in vitro and in vivo results indicate that SA‐Co@MoS_2_ nanoflowers can significantly eliminate tumor cells. This study offers valuable insights into the exploration of single‐atom doping‐enhanced piezoelectric sonosensitizers for cancer treatment, potentially paving the way for advancements in the field of piezocatalytic synergistic enzyodynamic therapy.

## Graphical abstract

The single‐atom doped molybdenum disulfide piezocatalyst markedly enhances both piezoelectric and enzyme‐mimicking activities, accelerating glutathione depletion. It induces mitochondrial dysfunction and cell cycle arrest, ultimately driving apoptosis while inactivating glutathione peroxidase 4 to initiate ferroptosis. This study establishes a novel paradigm for single‐atom doping‐enhanced piezoelectric sonosensitizers, marking a substantial advancement in the field of piezocatalytic synergistic enzydynamic tumor treatment.

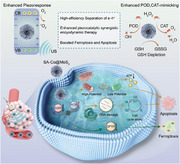

## 1 Introduction

Cancer remains a significant threat to human health due to its high incidence and mortality rates.^[^ ^1^ ^]^ Conventional chemotherapies face critical challenges, including systemic toxicity and the development of drug resistance.^[^ ^2^ ^]^ Consequently, there is an urgent need for alternative therapeutic strategies that effectively target the complex mechanisms underlying cancer pathogenesis. Reactive oxygen species (ROS) are pivotal in regulating redox the balance within tumor tissues, making them essential components in cancer therapy.^[^ ^3^ ^]^ The accumulation of excessive ROS disrupts tumor homeostasis, leading to lipid peroxidation, protein denaturation, and DNA damage, ultimately triggering apoptosis or necrosis.^[^ ^4^ ^]^ In recent years, numerous nanomedicine‐enabled treatment modalities have emerged that leverage ROS overproduction in tumor tissues, induced by either exogenous or endogenous activators acting on nanosensitizers.^[^ ^5^ ^]^ These modalities are designed to generate reactive radicals, facilitating efficient disease‐targeted therapies with reduced side effects and enhanced specificity. Among these approaches, photodynamic therapy, sonodynamic therapy, chemodynamic therapy, and radiodynamic therapy have gained significant recognition as promising strategies for cancer treatment. These innovative techniques capitalize on the unique properties of nanomaterials to enhance therapeutic efficacy while minimizing harm to healthy tissues.

The resurgence of piezoelectric electronics has revealed promising applications in mechanically vibration‐mediated tumor treatments through piezoelectric semiconductor nanomaterials, known as piezocatalytic therapy.^[^ ^6^ ^]^ Under mechanical forces like ultrasound (US) stimulation, the piezoelectric materials undergo lattice deformation, enhancing the separation and migration of electron‐hole pairs to the surface, thereby inducing redox reactions and generating ROS.^[^ ^7^ ^]^ Various piezocatalysts, such as BaTiO_3_,^[^ ^8^ ^]^ WS_2_,^[^ ^9^ ^]^ BiO_2‐x_,^[^ ^10^ ^]^ ZnO,^[^ ^11^ ^]^ and CaBi_2_Nb_2_O_9_,^[^ ^12^ ^]^ have emerged as candidates for cancer therapy. However, clinical translation faces challenges such as suboptimal piezoelectric responses, rapid electron‐hole recombination, inefficient energy harvesting, and unclear active sites. Some optimization measures, including phase engineering,^[^ ^13^ ^]^ crystal facet regulation,^[^ ^14^ ^]^ vacancy defects,^[^ ^15^ ^]^ doping engineering,^[^ ^16^ ^]^ heterostructure construction,^[^ ^17^ ^]^ and polarization enhancement,^[^ ^18^ ^]^ have been employed to enhance piezoelectric catalytic performance. Phase engineering facilitates easier electric domain deflection in coexisting phase regions, enhancing piezoelectric polarity.^[^ ^19^ ^]^ Crystal facet regulation exposes high‐energy crystal facets, which improve affinity, charge carrier separation, and active sites.^[^ ^20^ ^]^ Vacancy defects and doping engineering lead to lattice distortion, increased polarization, adjusting electronic structure, and accelerating dynamic transport of charge carriers.^[^ ^15^, ^16^ ^]^ The formation of heterojunctions is driven by energy band differences between various semiconductors, where the interface created by contact exhibits a potential difference, generating an internal electric field.^[^ ^21^ ^]^ Furthermore, the initial charge on the surface of piezoelectric semiconductors and the tilted energy bands can accelerate redox reactions at the heterojunction interface. Increased polarization from crystal growth in specific directions results in a greater piezoelectric potential, effectively boosting piezoelectric catalysis.^[^ ^22^ ^]^ These advancements indicate that key challenges facing piezoelectric materials can be addressed, unlocking their immense potential in biomedical applications, particularly in cancer treatment.

Besides exogenous activators, recent studies emphasize the importance of utilizing endogenous stimuli within the tumor microenvironment (TME) to amplify intratumoral oxidative stress, thereby enhancing the efficacy of ROS‐mediated cancer treatments.^[^ ^23^ ^]^ Nanozymes, artificial catalysts that exhibit natural enzyme‐mimicking behavior, have emerged as effective agents capable of converting intracellular substances into highly toxic ROS, amplifying antitumor effects.^[^ ^24^ ^]^ Nonetheless, the limited affinity of these nanozymes for hydrogen peroxide (H_2_O_2_), coupled with a shortage of endogenous H_2_O_2_, poses considerable challenges to the efficacy of tumor treatments.^[^ ^25^ ^]^ Several factors, including morphology and size,^[^ ^26^ ^]^ vacancies engineering,^[^ ^27^ ^]^ heteroatom doping,^[^ ^28^ ^]^ and surface modifications,^[^ ^29^ ^]^ play critical roles in determining the catalytic activity and substrate selectivity of nanozymes. In particular, alterations in morphology and size can lead to changes in the exposed crystal facets, crystal structure, and specific surface area of the nanocatalysts, impacting their catalytic performance.^[^ ^30^ ^]^ Furthermore, the incorporation of heteroatoms and the engineering of vacancies precisely modulate catalytic activity by adjusting the surface electronic properties and energy band structures of the nanozymes.^[^ ^24^, ^31^ ^]^ Additionally, surface modifications can further enhance catalytic efficiency by affecting the number of active sites, the affinity for substrates, and the overall physicochemical characteristics of the nanozymes.^[^ ^32^ ^]^ Moreover, glutathione (GSH), a key intracellular antioxidant, mitigates oxidative damage by scavenging ROS, thereby reducing the effectiveness of ROS‐based therapies.^[^ ^33^ ^]^ Thus, strategies aimed at improving oxidative stress and depleting GSH are a promising avenue to potentiate antitumor responses.

**Scheme 1:**
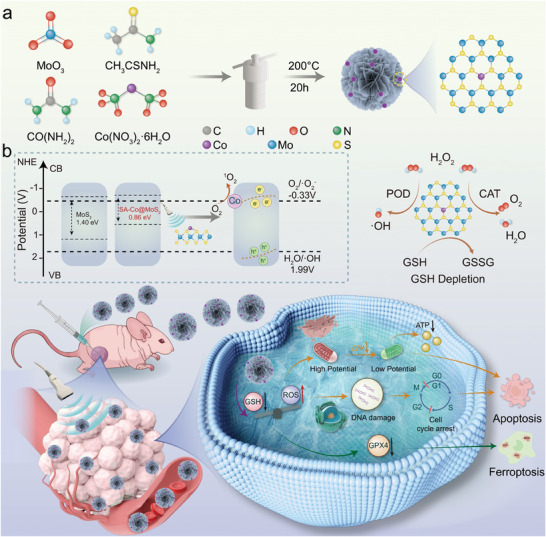
Schematic illustration of SA‐Co@MoS_2_ nanoflowers engineered for synergistic antitumor therapy, combining US‐enhanced piezoelectric catalysis and enzymatic activity. a) The synthetic procedure of SA‐Co@MoS_2_ nanoflowers. b) Diagram illustrating the SA‐Co@MoS_2_ nanoflowers as TME modulators and their mechanisms in disrupting redox homeostasis, leading to apoptosis and ferroptosis (Created by Huiyantong Animation Technology Co., Ltd).

Molybdenum disulfide (MoS_2_) is a promising candidate for piezoelectric materials because of its non‐centrosymmetric structure.^[^ ^34^ ^]^ Few‐layer or monolayer MoS_2_ exhibits a strong piezoelectric response.^[^ ^35^ ^]^ Leveraging this property, researchers have synthesized flower‐like MoS_2_ with abundant edge sites specifically for the piezocatalytic degradation of organic dyes.^[^ ^36^ ^]^ Additionally, MoS_2_ demonstrates negligible toxicity, enabling its widespread application in biological fields.^[^ ^37^ ^]^ Single‐atom doping is an innovative strategy used to improve catalytic performance, inducing out‐of‐plane polarization, leading to a high density of vacancies and a reduced bandgap, ultimately enhancing carrier separation and movement.^[^ ^38^ ^]^ The vacancies serve as electron traps, reducing the recombination of electrons and holes while acting as active sites for substrate molecules such as water (H_2_O) and oxygen (O_2_), facilitating catalytic reactions.^[^ ^39^ ^]^ Herein, we present cobalt (Co) single‐atom doped MoS_2_ (SA‐Co@MoS_2_) nanoflowers designed to enhance both piezoelectric and enzymatic catalysis for augmented cancer therapy. SA‐Co@MoS_2_ nanoflowers were synthesized via a facile solvothermal method (**Scheme** **1**a), optimizing the electronic band structures and increasing sulfur vacancies to enhance ROS generation and GSH depletion. Co single‐atom doping effectively delays electron‐hole recombination, improving charge separation and migration under US irradiation (Scheme 1b). Additionally, the MoS_2_ nanoflowers act as a co‐catalyst by efficiently reducing Co^3+^ to Co^2+^ at the exposed Mo^4+^ active sites, sustaining the Fenton‐like reaction. The SA‐Co@MoS_2_ nanoflowers exhibit notable catalase (CAT)‐mimicking activity, decomposing H_2_O_2_ into O_2,_ and alleviating tumor hypoxia. Furthermore, Co^2+^ facilitates GSH depletion, impairing the ability of tumor cells to neutralize ROS. The resulting increase in intracellular ROS triggers mitochondrial dysfunction, cell cycle arrest, and ferroptosis, ultimately inducing tumor cell death. These findings underscore the potential of SA‐Co@MoS_2_ nanoflowers as a robust platform for synergistic piezocatalytic and enzyodynamic cancer therapy.

## 2 Results and Discussion

### 2.1 Characterization of SA‐Co@MoS_2_ Nanoflowers

**Figure 1:**
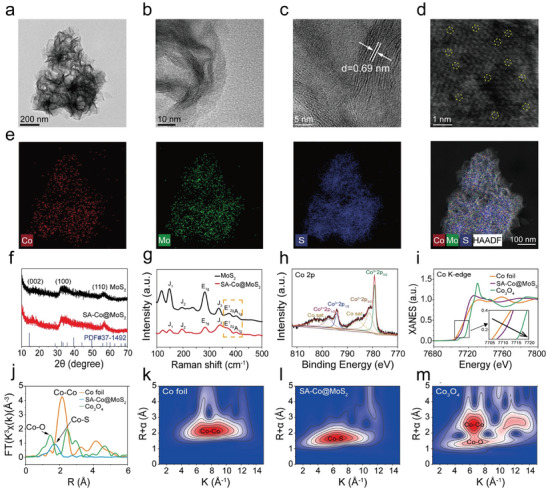
Morphology and structural characterization of the SA‐Co@MoS_2_ nanoflowers. a,b) TEM images of SA‐Co@MoS_2_ nanoflowers. c) HRTEM image of SA‐Co@MoS_2_ nanoflowers. d) Spherical aberration‐corrected TEM image of SA‐Co@MoS_2_ nanoflowers. e) EDS element mapping of SA‐Co@MoS_2_ nanoflowers. f) XRD patterns and g) Raman spectra of MoS_2_ and SA‐Co@MoS_2_ nanoflowers. h) Co 2p XPS spectra of SA‐Co@MoS_2_ nanoflowers. i) Co K‐edge XANES spectra of Co foil, Co_3_O_4_, and SA‐Co@MoS_2_ nanoflowers (inset is the magnified image). j) FT and k–m) WT of SA‐Co@MoS_2_ nanoflowers and reference samples.

The SA‐Co@MoS_2_ nanoflowers were synthesized using a straightforward one‐pot hydrothermal method (Scheme 1a). Transmission electron microscopy (TEM) images reveal the characteristic nanoflower morphology of MoS_2_, with Co doping showing no significant alterations in structural features (**Figure** **1**a; Figure S1a, Supporting Information). High‐resolution TEM (HRTEM) images further confirm that both MoS_2_ and SA‐Co@MoS_2_ nanoflowers consist of monolayer and few‐layer structures at their edges, displaying crystal asymmetry linked to piezoelectric properties (Figure 1b; Figure S1b, Supporting Information). The measured lattice stripe spacings for MoS_2_ and SA‐Co@MoS_2_ nanoflowers are 0.62 and 0.69 nm, respectively, corresponding to the (002) plane of MoS_2_ nanoflowers (Figure 1c; Figure S1c, Supporting Information). Importantly, high‐angle annular dark‐field scanning transmission electron microscopy (HAADF‐STEM) images reveal the atomic dispersion of Co atoms, appearing as bright spots within the MoS_2_ nanoflowers lattice, accompanied by structural defects (Figure 1d; Figure S2, Supporting Information). Energy‐dispersive X‐ray spectroscopy (EDS) analysis indicates a uniform distribution of Co, Mo, and S elements across the nanoflowers (Figure 1e), with the Co amount determined as 4.86 wt.% using inductively coupled plasma optical emission spectroscopy (ICP‐OES). X‐ray diffraction (XRD) analysis reveals that Co doping does not significantly affect the crystalline structure of MoS_2_ nanoflowers (Figure 1f), as evidenced by the consistent diffraction peaks with pristine MoS₂ nanoflowers and the absence of metallic Co or oxide phases. The peaks at 14.4, 33.0, and 58.3° correspond to the (002), (100), and (110) planes of MoS_2_ nanoflowers, respectively, consistent with the JCPDS card no.37‐1492. Raman spectroscopy reveals peaks at 374.1 and 401.1 cm⁻¹, corresponding to the E^1^_2g_ and A_1g_ vibrational modes, thereby confirming the successful synthesis of the semiconductor phase (Figure 1g). However, Co doping results in peak broadening and a reduction in intensity, suggesting disruptions in Mo‐S vibrational modes, enhanced edge exposure, and the presence of structural defects. High‐resolution X‐ray photoelectron spectroscopy (XPS) analysis further validates Co doping, showing peaks corresponding to Co, Mo, and S atoms (Figure 1h; Figure S3, Supporting Information). The Mo 3d XPS spectra reveal characteristic peaks at 228.6 and 231.8 eV corresponding to the Mo^4+^ 3d_5/2_ and Mo^4+^ 3d_3/2_, along with additional peaks at 232.7 and 235.5 eV for Mo^6+^ 3d_5/2_ and Mo^6+^ 3d_3/2_, respectively (Figures S4 and S5, Supporting Information). A shift of ≈0.13 eV toward lower binding energy in the Mo peaks of SA‐Co@ MoS_2_ nanoflowers, indicating an increase in electronic density (Figure S6, Supporting Information), contributes to the observed enhancements in enzymatic catalytic activity and piezoelectric performance. Quantitative XPS analysis indicates that Co^2+^ species account for 59.9% of the Co present in the SA‐Co@MoS_2_ nanoflowers.

X‐ray absorption near edge structure (XANES) and extended X‐ray absorption fine structure (EXAFS) spectroscopy were employed to further investigate the coordination environment and valence states of Co in SA‐Co@MoS_2_ nanoflowers. The XANES Co K‐edge spectrum positioned between Co_3_O_4_ and Co foils indicates that the average valence state of Co lies between 0 and +3 (Figure 1i), consistent with the XPS results. The Fourier transform (FT) of the EXAFS spectrum in R‐space reveals a Co‐S peak at 1.71 Å and the absence of a Co─Co peak at 2.14 Å (Figure 1j). Fitting of EXAFS data shows an average Co─S bond length of 2.21 Å and a coordination number of 2.8 for Co atoms (Figures S7–S9 and Table S1, Supporting Information). Wavelet transform (WT) EXAFS analyses confirm the absence of Co─Co bonds and the successful incorporation of Co as isolated single atoms within the MoS_2_ nanoflowers structure, affirming the formation of atomically dispersed SA‐Co@MoS_2_ nanoflowers (Figure 1k–m).

Dynamic light scattering (DLS) measurements demonstrate similar average hydrodynamic diameters of MoS_2_ and SA‐Co@MoS_2_ nanoflowers, ≈328 and 331 nm, respectively (Figure S10, Supporting Information). Further analysis across various physiological solutions confirms that the surrounding environment has minimal impact on particle size (Figure S11, Supporting Information). Zeta potential measurements reveal values of −23.1 and −27.9 mV for MoS_2_ and SA‐Co@MoS_2_ nanoflowers, respectively (Figure S12, Supporting Information). Moreover, stability tests show excellent dispersion and colloidal stability in PBS for 7 days (Figure S13, Supporting Information).

### 2.2 Piezocatalytic Mechanism of SA‐Co@MoS_2_ Nanoflowers

**Figure 2:**
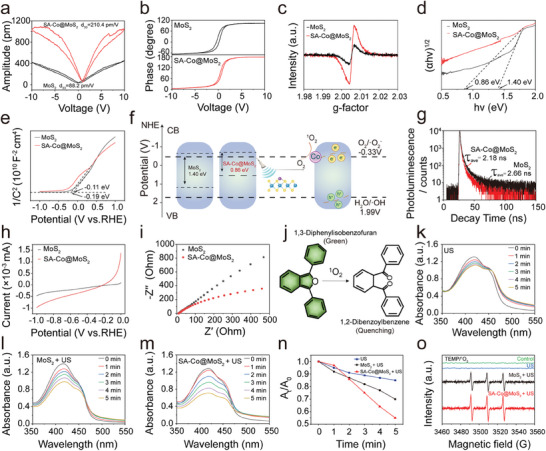
Piezoelectric performance of SA‐Co@MoS_2_ nanoflowers. a) Amplitude‐voltage curves and b) phase hysteresis loops of both MoS_2_ and SA‐Co@MoS_2_ nanoflowers. c) EPR spectra, d) band gap measurements, and e) Mott‐Schottky plots of both MoS_2_ and SA‐Co@MoS_2_ nanoflowers. f) Schematic illustration of band tilting induced by US‐driven pressure and the accompanied redox reaction. g) TRPL, h) LSV, and i) EIS spectra of both MoS_2_ and SA‐Co@MoS_2_ nanoflowers. j) The reaction mechanism between DPBF and ^1^O_2_. UV‐vis absorption spectra of DPBF solutions incubated with k) H_2_O, l) MoS_2_, and m) SA‐Co@MoS_2_ nanoflowers under US irradiation (1.0 MHz, 1.0 W cm^−2^, 50% duty cycle) over different time intervals. n) The oxidation rates of DPBF in solutions of H_2_O, MoS_2_, and SA‐Co@MoS_2_ nanoflowers under US irradiation (1.0 MHz, 1.0 W cm^−2^, 50% duty cycle) over times. o) ESR spectra of the production of ^1^O_2_ radicals in solutions of H_2_O, MoS_2_, and SA‐Co@MoS_2_ nanoflowers under US irradiation (1.0 MHz, 1.0 W cm^−2^, 50% duty cycle) using TEMP a spin‐trapping agent.

To evaluate the piezoelectric properties of the synthesized materials, piezo‐response force microscopy (PFM) was employed to characterize both MoS₂ and SA‐Co@MoS_2_ nanoflowers (**Figure** **2**a,b). Under an applied bias voltage ranging from −10 to + 10 V, the materials exhibit classic piezoelectric signatures, including amplitude butterfly curves and phase hysteresis loops with a 180° phase reversal, confirming piezoelectric behavior. The piezoelectric coefficient (d_33_), a critical parameter for assessing the capacity of piezoelectric materials to convert mechanical energy into electrical energy, was measured. The d_33_ values for MoS_2_ and SA‐Co@MoS_2_ nanoflowers are determined to be 88.2 and 210.4 pm v^−1^, respectively, as derived from the amplitude butterfly loops. This substantial enhancement in the piezoelectric response of MoS_2_ nanoflowers can be attributed to the incorporation of single Co atoms. Further electron paramagnetic resonance (EPR) analyses reveal the presence of sulfur vacancies in SA‐Co@MoS_2_ nanoflowers, with a distinct signal peak at g = 2.004 (Figure 2c). These vacancies are crucial, serving as electron‐hole pair trapping sites, suppressing recombination, and promoting carrier transport, which collectively contributes to the enhanced piezoelectric performance of SA‐Co@MoS_2_ nanoflowers.

To gain deeper insights into the electronic properties, the band structures of materials were investigated. The band gaps of MoS_2_ and SA‐Co@MoS_2_ nanoflowers are determined to be 1.40 and 0.86 eV, respectively, using Tauc plots derived from the Kubelka‐Munk function (Figure 2d; Figure S14, Supporting Information). Additionally, the flat band potentials are calculated using Mott‐Schottky plots (Figure 2e). Both materials exhibit positive slopes, consistent with n‐type semiconductor behavior. The flat band potentials (E_fb_) relative to the reversible hydrogen electrode (RHE) were calculated using the following equation:

(1) $$E_{\mathrm{fb}}\;\;\left(\mathrm{vs}\;\mathrm{RHE}\right)=E_{\mathrm{fb}}\left(\mathrm{vs}\;\mathrm{NHE}\right)+0.059\;\times \;\mathrm{pH}$$

where the pH of the Na_2_SO_4_ electrolyte is 6.8, and the E_fb_ relative to the normal hydrogen electrode (NHE) is determined to be −0.51 and −0.59 V for MoS_2_ and SA‐Co@MoS_2_ nanoflowers, respectively. As the conduction band (CB) of n‐type semiconductors is typically 0.1 V lower than the E_fb_, the CB for MoS_2_ and SA‐Co@MoS_2_ nanoflowers was calculated to be −0.61 and −0.69 V, respectively. Using the measured band gaps, the valence band (VB) potentials for MoS_2_ and SA‐Co@MoS_2_ nanoflowers were estimated to be 0.79 and 0.17 V, respectively (Figure 2f). To investigate the carrier dynamics, time‐resolved photoluminescence (TRPL) spectroscopy was performed, providing valuable insights into charge separation and recombination process in MoS_2_ and SA‐Co@MoS_2_ nanoflowers (Figure 2g). The fluorescence lifetime curves were fitted using a double‐exponential decay model, as described by the equation (Table S2, Supporting Information):

(2) $$I\left(t\right)=B_{1}e^{-t/\tau 1}+B_{2}e^{-t/\tau 2}$$

where I(t) represents the fluorescence intensity, and short fluorescence lifetime (τ_1_) is the average time conduction electrons in a semiconductor stay in the conduction band before quickly recombining radiatively with holes. The long fluorescence lifetime (τ_2_) represents the time electrons transfer energy to the valence band and recombine without emitting photons. B_1_ and B_2_ are the weighting factors. The average fluorescence lifetime (τ_ave_) is the weighted average of τ_1_ and τ_2_, and is calculated using the following equation:

(3) $$\tau_{\mathrm{ave}}=B_{1}{\tau_{1}}^{2}+B_{2}{\tau_{2}}^{2}/B_{1}\tau_{1}+B_{2}\tau_{2}$$

For MoS_2_ and SA‐Co@MoS_2_ nanoflowers, τ_1_ is determined to be 1.96 and 1.70 ns, while τ_2_ is 11.6 and 10.4 ns, respectively. The calculated τ_ave_ is 2.66 ns for MoS_2_ nanoflowers and 2.18 ns for SA‐Co@MoS_2_ nanoflowers. The shorter lifetime of SA‐Co@MoS_2_ nanoflowers indicates enhanced carrier transfer efficiency and more effective electron‐hole separation. Furthermore, the significantly reduced photoluminescence (PL) intensity of SA‐Co@MoS_2_ nanoflowers compared to MoS_2_ nanoflowers further supports the conclusion that Co doping suppresses electron‐hole pairs recombination (Figure S15, Supporting Information). Charge carrier dynamics were further examined using a linear scanning voltammogram (LSV) (Figure 2h). Upon an applied electrical potential, SA‐Co@MoS_2_ nanoflowers exhibit a significant increase in current density, indicating improved charge transport properties. Electrochemical impedance spectroscopy (EIS) was conducted to evaluate carrier transfer efficiency, where the arc radius in the Nyquist plot represents charge transfer resistance (Figure 2i). The smaller arc radius observed for SA‐Co@MoS_2_ nanoflowers compared to MoS_2_ nanoflowers reflects lower charge transfer resistance, confirming more efficient carrier transport. These findings suggest that Co doping increases sulfur vacancies modifies the band structure, enhances electron‐hole pair separation, and accelerates carrier transfer, ultimately improving ROS generation efficiency.

The generation of singlet oxygen (^1^O_2_) was assessed using 1,3‐diphenylisobenzofuran (DPBF) as a probe. In the presence of ^1^O_2_, DPBF undergoes irreversible oxidation, resulting in the decomposition of 1,2‐dibenzoylbenzene and a subsequent rapid decrease in absorbance at 421 nm, as evidenced in the ultraviolet‐visible (UV‐vis) absorption spectrum (Figure 2j). We synthesized MoS₂ nanoflowers doped with varying amounts of Co, designated as SA‐Co@MoS_2_‐5, SA‐Co@MoS_2_‐10, and SA‐Co@MoS_2_‐15. The amount of ROS generated was evaluated by monitoring the reduction of DPBF after 5 min of US treatment and the increase in 3,3′,5,5′‐tetramethylbenzidine (TMB) after 5 min of reaction with H₂O₂ (Figure S16, Supporting Information). The findings indicated that SA‐Co@MoS_2_‐5 displayed the highest ROS production ability. Single‐atom doping increases the number of available active sites, which enhances sonodynamic and catalytic reactions. However, excessively high doping concentrations lead to stronger interactions between active sites, which promote electron‐hole recombination and reduce carrier density, thereby diminishing both sonodynamic and catalytic activities. Overall, the optimal doping amount for SA‐Co@MoS_2_ is 5 mg, which demonstrates excellent piezoelectric response and catalytic effects. Therefore, we chose SA‐Co@MoS_2_‐5 as the representative model for the following experiments. In the control group, the absorption peak of DPBF at 421 nm exhibits only a minimal decrease after US exposure for 5 min, indicating limited ^1^O_2_ generation. In contrast, both MoS_2_ and SA‐Co@MoS_2_ nanoflowers display a pronounced time‐dependent decrease in absorbance upon US irradiation, with SA‐Co@MoS_2_ nanoflowers demonstrating a significantly greater reduction. This enhanced decrease in absorbance is indicative of improved ROS generation attributed to single‐atom Co doping (Figure 2k–n). To investigate the piezoelectric catalytic performance of MoS_2_ and SA‐Co@MoS_2_ nanoflowers, electron spin resonance (ESR) measurements were conducted using 2,2,6,6‐tetramethylpiperidine (TEMP) as a spin‐trapping agent for ^1^O_2_ (Figure 2o). Notably, SA‐Co@MoS_2_ nanoflowers exhibit a markedly higher intensity of the ^1^O_2_ signal compared to MoS_2_ nanoflowers under US irradiation, corroborating the enhanced generation of ROS in the doped nanoflowers. These findings clearly demonstrate that the incorporation of single Co atoms into MoS_2_ nanoflowers significantly enhances their ability to generate ROS under US irradiation, thereby improving their piezoelectric catalytic performance.

### 2.3 GSH Consumption and Enzyme‐Mimicking Activities of SA‐Co@MoS_2_ Nanoflowers

**Figure 3:**
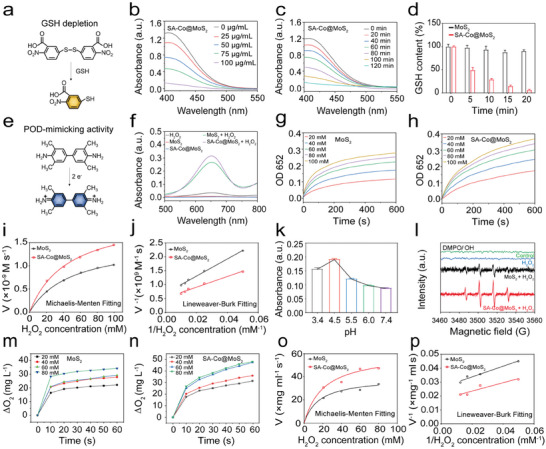
GSH depletion and enzyme‐mimicking activities of SA‐Co@MoS_2_ nanoflowers. a) Schematic diagram of the DTNB reaction mechanism. GSH consumption of SA‐Co@MoS_2_ nanoflowers using DTNB assay at different concentrations b) and at different time points c). d) Time‐dependent change in absorbance at 412 nm after incubation of MoS_2_ and SA‐Co@MoS_2_ nanoflowers with GSH solution (*n* = 3). e) Schematic representation of the POD‐mimicking catalytic mechanism of MoS_2_ and SA‐Co@MoS_2_ nanoflowers. f) UV‐vis spectra of different groups incubated with H_2_O_2_ for 10 min. Enzyme kinetic profiles of g) MoS_2_ and h) SA‐Co@MoS_2_ nanoflowers treated with different H_2_O_2_ concentrations. i) Michaelis‐Menten kinetics and j) Lineweaver‐Burk plot for POD‐mimicking activities of MoS_2_ and SA‐Co@MoS_2_ nanoflowers_._ k) POD‐mimicking activities of SA‐Co@MoS_2_ nanoflowers at different pH values (*n* = 3). l) ESR spectra of the ·OH production by MoS_2_ and SA‐Co@MoS_2_ nanoflowers incubated with H_2_O_2_ using DMPO as a spin‐trapping agent. O_2_ production after incubation with m) MoS_2_ and n) SA‐Co@MoS_2_ with H_2_O_2_ at different concentrations. o) Michaelis‐Menten kinetics and p) Lineweaver‐Burk plot for CAT‐mimicking activities of MoS_2_ and SA‐Co@MoS_2_ nanoflowers.

GSH serves as a critical antioxidant, effectively scavenging ROS. Thus, a strategy that depletes GSH while simultaneously increasing ROS production offers a promising approach to enhance therapeutic efficacy. To assess GSH consumption, 5,5′‐ dithiobis (2‐nitrobenzoic acid) (DTNB) was utilized as a probe. The mechanism involves the reduction of colorless DTNB by GSH to form a yellow product (**Figure** **3**a). As the concentration of SA‐Co@MoS_2_ nanoflowers increases and the incubation time extends, a corresponding decrease in absorbance at 412 nm is observed, indicating effective consumption of GSH in solution (Figure 3b,c). In contrast, MoS_2_ nanoflowers alone cannot significantly deplete GSH, while SA‐Co@MoS_2_ nanoflowers completely exhaust GSH within 20 min, which is attributed to oxidation by Co (Figure 3d; Figures S17and S18, Supporting Information). We performed a quantitative analysis of the Co elemental valence state distribution before and after the reaction of SA‐Co@MoS_2_ nanoflowers with GSH (Figure S19, Supporting Information). XPS analysis revealed a significant decrease in Co^2+^ content, dropping from 59.9% to 30.9% after reaction with GSH. In contrast, the Co^3+^ content showed a marked increase, rising from 40.1% to 79.1%. This substantial change in valence states strongly confirms that Co^2+^ in SA‐Co@MoS_2_ nanoflowers was effectively oxidized to Co^3+^ during the interaction with GSH, accompanied by the reduction of GSH.

Given the elevated concentrations of H_2_O_2_ in TME, potentially reaching up to 100 µm, peroxidase (POD)‐mimicking activity of SA‐Co@MoS_2_ nanoflowers was assessed using TMB, which reacts with hydroxyl radical (•OH) to yield a blue product with a peak absorption at 652 nm (Figure 3e). Notably, SA‐Co@MoS_2_ nanoflowers exhibit superior POD‐mimicking activity compared to MoS_2_ nanoflowers (Figure 3f). Enzyme kinetics analyses reveal that SA‐Co@MoS_2_ nanoflowers (2.11 × 10^−9^ m s^−1^) demonstrate a greater affinity for H_2_O_2_ and higher maximum velocities (V_max_) compared to MoS_2_ nanoflowers (1.53 × 10^−9^ m s^−1^) (Figure 3g–j). The POD‐mimicking activity triggered by SA‐Co@MoS_2_ nanoflowers was analyzed using XPS after the reaction with H_2_O_2_ (Figure S20, Supporting Information). The results showed that Co^2+^ and Co^3+^ accounted for 54.3% and 45.7%, respectively, of the total Co content in the nanoflowers, which is nearly identical to the proportion of Co before the reaction. Additionally, a stronger peak was observed at 235.7 eV in the Mo 3d XPS spectrum of the SA‐Co@MoS_2_ nanoflowers, corresponding to the Mo^6+^ species, with an abundance of ≈14.9%. In contrast, in the original SA‐Co@MoS_2_ nanoflowers, although a peak at the same binding energy was observed, its intensity was weaker, and the Mo^6+^ content was only ≈9.3%. These findings strongly confirm that, during the POD‐mimicking reaction, Mo^4+^ was oxidized to Mo^6+^. Additionally, SA‐Co@MoS_2_ nanoflowers display pH‐dependent POD‐mimicking activity (Figure 3k). Spin‐trapping experiments employing 5,5‐dimethyl‐1‐pyrroline N‐oxide (DMPO) confirm the enhanced production of •OH due to single‐atom doping (Figure 3l). Both the SA‐Co@MoS_2_ + H_2_O_2_ and the MoS_2_ + H_2_O_2_ groups exhibit characteristic quartet signals, consistent with prior enzyme kinetics findings. Moreover, the SA‐Co@MoS_2_ + H_2_O_2_ group displays an enhanced •OH signal.

The CAT‐mimicking activity of SA‐Co@MoS_2_ nanoflowers was investigated, which is crucial for decomposing excess H_2_O_2_ into O_2_ (Figure S21, Supporting Information), thereby alleviating tumor hypoxic conditions. The SA‐Co@MoS_2_ nanoflowers demonstrate a higher dissolved O_2_ content and produce more O_2_ bubbles compared to MoS_2_ nanoflowers, with increases observed that correlate with both incubation time and H_2_O_2_ concentration (Figure 3m,n). Michaelis‐Menten kinetic analyses reveal that SA‐Co@MoS_2_ nanoflowers (1.01 mg mL^−1^ s^−1^) exhibit a higher V_max_ than MoS_2_ nanoflowers (0.65 mg mL^−1^ s^−1^) (Figure 3o,p).

### 2.4 Enhanced Piezoelectric and Catalytic Mechanism Revealed by Density Functional Theory Calculation

**Figure 4:**
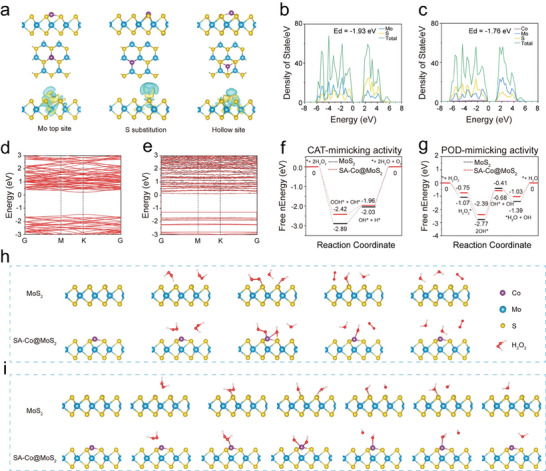
DFT calculations and enhanced catalytic mechanism. a) Top views, side views, and corresponding differential charge density of the geometries for Co on the Mo top site, S substitution, and the hollow site of MoS_2_ nanoflowers. DOS profiles of b) MoS_2_ and c) SA‐Co@MoS_2_ nanoflowers. Electronic band structures of d) MoS_2_ and e) SA‐Co@MoS_2_ nanoflowers. Corresponding free energy diagrams for f) CAT‐ and g) POD‐mimicking activities on MoS_2_ and SA‐Co@MoS_2_ nanoflowers. Catalytic mechanisms for h) CAT‐ and i) POD‐mimicking activities of MoS_2_ and SA‐Co@MoS_2_ nanoflowers.

To elucidate the mechanism underlying the enhanced piezoelectric and catalytic effects from single‐atom Co doping, we performed density functional theory (DFT) calculations. Geometric optimization of SA‐Co@MoS_2_ nanoflowers was conducted to evaluate various adsorption sites for the Co atom, specifically the Mo‐top (Co atom is located above the Mo atom), S‐site (Co atom replaces the position of the S atom), and hollow site (Co atom is located at the center of the hexagonal crystal system) (**Figure** **4**a). The calculation reveals that the Co atom exhibits the highest stability when adsorbed at the Mo‐top site, with a binding energy of ‐2.94 eV, significantly greater than that at the hollow site (−2.65 eV) and S‐site (−1.44 eV). Structural analyses indicate that Co doping leads to elongation of Mo─S bonds and reduction in bond angles, consistent with the lattice distortions observed in HAADF‐STEM (Figure S2 and Table S3, Supporting Information). Additionally, differential charge mappings illustrate substantial electron accumulation around the Co atom at the Mo‐top site, disrupting the original charge distribution symmetry of MoS_2_ nanoflowers (Figure 4a). Based on Bader charge analysis, the electron loss of Mo at the Mo‐top, S‐substituted, and hollow sites are 1.03e, 0.64e, and 0.77e, respectively. Furthermore, a comparison analysis of the density of states (DOS) and band structures between pristine MoS₂ and SA‐Co@MoS_2_ nanoflowers reveals that Co‐doping significantly alters the electronic properties of MoS_2_ nanoflowers. Notably, the d‐band center of SA‐Co@MoS_2_ nanoflowers shifts closer to the Fermi level at −1.76 eV compared to −1.93 eV in MoS_2_ nanoflowers, indicating enhanced interactions with substrates that are favorable for catalytic processes (Figure 4b,c). Moreover, the bandgap is narrowed from 1.81 to 1.37 eV, facilitating easier electron‐hole pair generation and thereby improving the piezoelectric performance (Figure 4d,e).

To investigate the origins of the enhanced enzymatic activity, we calculated the Gibbs free energy changes associated with CAT‐ and POD‐mimicking reactions for both MoS_2_ and SA‐Co@MoS_2_ nanoflowers. In the CAT‐mimicking reaction, two H_2_O_2_ molecules adsorb onto SA‐Co@MoS_2_ nanoflowers decomposed into OOH* and OH* intermediates, which subsequently react to release O_2_. The calculations demonstrate that SA‐Co@MoS_2_ nanoflowers exhibit a significantly lower energy barrier of 0.46 eV compared to 0.86 eV for MoS_2_ nanoflowers, confirming their superior capability for O_2_ generation (Figure 4f,h). For POD‐mimicking activity, the adsorption and cleavage of H_2_O_2_ into two OH* intermediates are more thermodynamically favorable on SA‐Co@MoS_2_ nanoflowers, with a lower energy barrier of 1.71 versus 2.36 eV for MoS_2_ nanoflowers, promoting the generation of •OH radicals (Figure 4g,i). After the reactions, the OH* intermediates desorb as H_2_O, allowing the catalyst to return to its initial state. These findings collectively demonstrate that Co doping effectively lowers the energy barriers of critical steps in the catalytic reactions, thereby enhancing the enzymatic activities of MoS_2_ nanoflowers.

### 2.5 In Vitro Antitumor Effect

**Figure 5:**
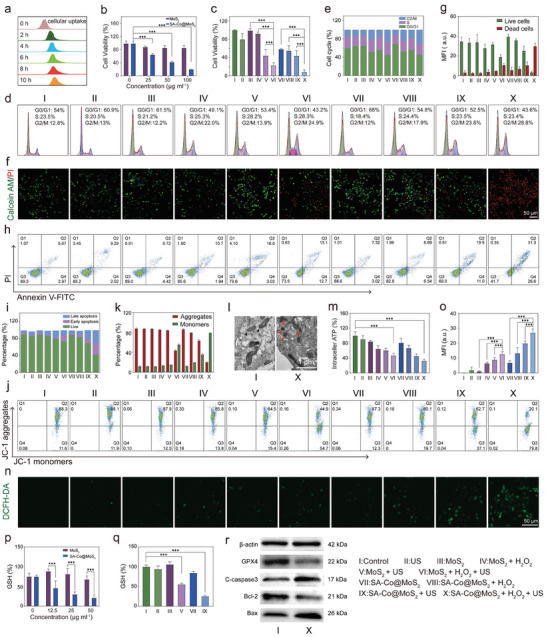
In vitro synergistic therapeutic effects of SA‐Co@MoS_2_ nanoflowers. a) Analysis of cellular uptake of SA‐Co@MoS_2_ nanoflowers via flow cytometry. b) Relative cell viability in HepG2 cells following 24 h treatment with varying concentrations of MoS_2_ and SA‐Co@MoS_2_ nanoflowers (*n* = 3). c) Relative cell viability in HepG2 cells after different treatments (*n* = 4). d) Cell cycle redistribution among HepG2 cells subjected to different treatments and e) the corresponding semi‐quantitative analyses of the cell cycle phases. f) CLSM images of HepG2 cells post‐treatment stained with PI and Calcein‐AM and g) the corresponding semi‐quantitative analyses of live and dead cells (*n* = 3). h) Flow cytometric analyses of HepG2 cells post‐treatment co‐stained with Annexin V‐FITC and PI and i) the corresponding semiquantitative analyses. j) Flow cytometry analyses of mitochondrial membrane potential in HepG2 cells following various treatments and k) the corresponding semi‐quantitative analysis of JC‐1 aggregates and monomers (*n* = 3). l) Bio‐TEM images of HepG2 cells after different treatments. m) Intracellular ATP levels in HepG2 cells after different treatments (*n* = 3). n) CLSM images of HepG2 cells stained with DCFH‐DA after different treatments and o) the corresponding semi‐quantitative analyses (*n* = 3). p) Intracellular GSH levels in HepG2 cells after treating with different concentrations of MoS_2_ and SA‐Co@MoS_2_ nanoflowers (*n* = 3). q) Intracellular GSH levels in HepG2 cells after different treatments (*n* = 3). r) Western blot analysis of GPX4, cleaved‐caspase3, Bcl‐2, and Bax in HepG2 cells. Results are presented as means ± SD. Statistical significance was calculated using a one‐way ANOVA. ***p < 0.001.

Building on the exceptional piezoelectric and catalytic properties of SA‐Co@MoS_2_ nanoflowers, we further evaluated their in vitro treatment efficacy. Initially, the cellular uptake of SA‐Co@MoS_2_ nanoflowers was confirmed using Bio‐TEM analyses of HepG2 cells following an 8 h treatment period, demonstrating effective endocytosis of SA‐Co@MoS_2_ nanoflowers (Figure S22, Supporting Information). Flow cytometry analyses of HepG2 cells incubated with fluorescein isothiocyanate (FITC)‐labeled SA‐Co@MoS_2_ nanoflowers at intervals of 0, 2, 4, 6, 8, and 10 h indicate a time‐dependent increase in fluorescence intensity, confirming successful internalization (**Figure** **5**a). In terms of cytotoxicity, MoS_2_ nanoflowers exhibit minimal effects, even at a concentration of 100 µg mL^−1^. Conversely, SA‐Co@MoS_2_ nanoflowers demonstrate a concentration‐dependent cytotoxicity, resulting in ≈ 80% cell death at the same concentration (Figure 5b). To assess their in vitro anticancer effects, we selected both MoS_2_ and SA‐Co@MoS_2_ nanoflowers at a concentration of 25 µg mL^−1^ using the standard cell counting kit‐8 (CCK‐8) assay, incorporating additional H_2_O_2_ to mimic the TME (Figure S23, Supporting Information). Following H_2_O_2_ treatment, both MoS_2_ and SA‐Co@MoS_2_ nanoflowers exhibit enhanced cytotoxicity compared to their effects in isolation. Next, we investigated the piezoelectric and enzyodynamic synergistic therapeutic effects of SA‐Co@MoS_2_ nanoflowers. The combination of H_2_O_2_ and US significantly reduces cell viability by ≈90%, in contrast to the relatively small effects observed with either treatment alone (Figure 5c). Importantly, SA‐Co@MoS_2_ nanoflowers consistently exhibit greater cytotoxicity than MoS_2_ nanoflowers, highlighting the efficacy of single‐atom doping.

Immunofluorescence staining of γ‐H2AX, a representative biomarker of DNA double‐strand breaks, was performed to assess the extent of DNA damage (Figure S24, Supporting Information). Almost no green fluorescence signals were observed in HepG2 cells treated with the Control, US, MoS₂ nanoflowers, and SA‐Co@MoS_2_ nanoflowers. However, when treated with H₂O₂ and US, a significant green fluorescence signal was detected. This result strongly indicates that, under US stimulation, MoS₂ and SA‐Co@MoS_2_ nanoflowers can induce notable DNA damage through the synergistic effects of the piezoelectric effect and enzyme kinetics‐based therapy. Notably, the SA‐Co@MoS_2_ nanoflower group exhibited the most intense green fluorescence signal, further highlighting its enhanced tumor‐killing ability. To assess the impact of different treatment groups on cell cycle progression, we performed propidium iodide (PI) staining followed by flow cytometry (Figure 5d,e). In the control group, the majority of cells are in the G0/G1 phase, accounting for 54.0%, with 23.5% in the S phase and only 12.8% in the G2/M phase. However, treatments with MoS_2_ or SA‐Co@MoS_2_ nanoflowers in conjunction with H_2_O_2_ and US result in increased percentages of cells in the G2/M phase, reaching 24.9% and 28.8%, respectively. Simultaneously, there are significant decreases in the proportions of cells in the G0/G1 phase, dropping to ≈43.2% and 43.6%, respectively. These results indicate that the treatment induces cell cycle arrest at the G2/M phase, contributing to cancer cell death. To visually assess the therapeutic effects at the cellular level, we employed calcein acetoxymethyl ester (Calcein‐AM) and PI staining, where dead cells emit red and live cells fluoresce green (Figure 5f,g). Compared to the control, US, and MoS_2_ nanoflowers groups, MoS_2_ nanoflowers combined with H_2_O_2_ or US exhibit minimal cell damage, while the SA‐Co@MoS_2_ nanoflowers combined with H_2_O_2_ and US lead to significant cell death, underscoring its enhanced tumor‐killing effect. Further analyses of the intracellular therapeutic effect of SA‐Co@MoS_2_ nanoflowers were conducted using membrane‐linked protein V‐ FITC and PI staining assays (Figure 5h,i). Individually exposing the cells to the US induces ≈10% apoptosis. The addition of H_2_O_2_ slightly increases apoptosis rates to 12.6% for MoS_2_ nanoflowers and 15.2% for SA‐Co@MoS_2_ nanoflowers. Following US stimulation, MoS_2_ and SA‐Co@MoS_2_ nanoflowers exhibit significantly elevated apoptosis rates at 19.0% and 30.5%, respectively. The highest apoptosis rate is observed in the SA‐Co@MoS_2_ nanoflowers when combined with H_2_O_2_ and US, reaching 57.9%, which is significantly higher than the 25.8% apoptosis rate observed in the MoS_2_ nanoflowers.

Given the association between apoptosis and mitochondrial dysfunction, the 5,5′,6,6′‐tetrachloro‐1,1′,3,3′‐tetraethylbenzimidazolium and carbocyanine iodine‐dide (JC‐1) probe was used to assess mitochondrial damage across different groups. In healthy mitochondria, JC‐1 aggregates, forming polymers within the mitochondrial matrix. In contrast, damaged mitochondria fail to aggregate due to diminished membrane potential, leading to monomer formation. Following H_2_O_2_ exposure, MoS_2_ and SA‐Co@MoS_2_ nanoflowers induce relatively low levels of JC‐1 monomers at 13.8% and 19.7%, respectively. US alone results in moderate JC‐1 monomer levels at 15.4% for MoS_2_ nanoflowers and 37.1% for SA‐Co@MoS_2_ nanoflowers. The combination of H_2_O_2_ and US leads to a significant increase to 79.8% in JC‐1 monomers for SA‐Co@MoS_2_ nanoflowers, indicating severe mitochondrial damage, compared to 54.7% for MoS_2_ nanoflowers (Figure 5j,k). Furthermore, Bio‐TEM images further reveal morphological alterations in mitochondria among the different treatment groups (Figure 5l). In the SA‐Co@MoS_2_ + H_2_O_2_ + US group, mitochondria exhibit fractured and dissolved cristae, alongside increased membrane density. Compared to the control and US groups, the addition of H_2_O_2_ slightly reduces the intracellular ATP levels by 14.5% for MoS_2_ nanoflowers and 18.8% for SA‐Co@MoS_2_ nanoflowers. Following US stimulation, the intracellular ATP levels decrease by 38.4% for MoS_2_ nanoflowers and 54.1% for SA‐Co@MoS_2_ nanoflowers. The combination of SA‐Co@MoS_2_ nanoflowers with H_2_O_2_ and US exhibits the most pronounced ATP suppression, reaching 68.3%, significantly higher than the 54.6% observed in the MoS_2_ nanoflowers (Figure 5m), indicating impaired mitochondrial metabolic function. To further investigate the underlying killing mechanism, intracellular ROS levels were measured using 2′,7′‐dichlorodihydrofluorescein diacetate (DCFH‐DA) as a probe, followed by semi‐quantitative analyses via ImageJ (Figure 5n,o). Minimal fluorescence intensity is observed in HepG2 cells treated with the US, MoS_2_, and SA‐Co@MoS_2_ nanoflowers. Co‐treatment with H_2_O_2_ and US significantly elevates ROS production in the SA‐Co@MoS_2_ nanoflowers compared to MoS_2_ nanoflowers.

Last, we investigated the intracellular depletion of GSH levels (Figure 5p,q). Treatment with varying concentrations of MoS_2_ and SA‐Co@MoS_2_ nanoflowers reveals a concentration‐dependent decrease in GSH levels, with enhanced depletion observed under US stimulation. The expression of ferroptosis‐related protein glutathione peroxidase 4 (GPX4) and apoptosis‐related proteins, including cleaved caspase‐3, Bcl‐2, and Bax, was also examined (Figure 5r). The significant downregulation of GPX4 in the SA‐Co@MoS_2_ + H_2_O_2_ + US group indicates effective induction of ferroptosis, while increased cleaved caspase‐3 and Bax expression, coupled with decreased Bcl‐2 levels, substantiate the occurrence of apoptosis in the treated cells.

### 2.6 Transcriptomics Analysis

**Figure 6:**
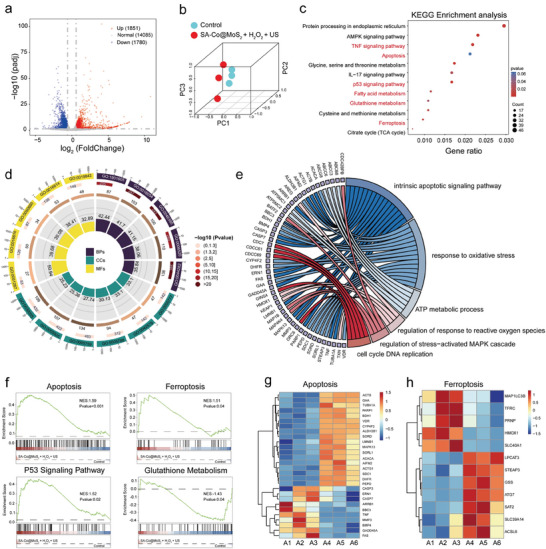
RNA sequencing analysis of HepG2 cells after different treatments. a) The volcano plot revealing the DEGs in the SA‐Co@MoS_2_ + H_2_O_2_ + US group relative to the control group. b) PCA analysis illustrating the β‐diversity among intratumor samples, effectively distinguishing the control group from the SA‐Co@MoS_2_ + H_2_O_2_ + US group. c) KEGG enrichment analysis showing the pathways enriched by DEGs in the SA‐Co@MoS_2_ + H_2_O_2_ + US group compared to the control group. d) The circular diagram representing the results of GO enrichment analysis. (GO:00 44282 small molecule catabolic process, GO:0 006520 cellular amino acid metabolic process, GO:00 46395 carboxylic acid catabolic process, GO:00 16054 organic acid catabolic process, GO:1 901 605 alpha‐amino acid metabolic process, GO:00 30055 cell‐substrate junction, GO:0 005925 focal adhesion, GO:0 005759 mitochondrial matrix, GO:0 005788 endoplasmic reticulum lumen, GO:00 42579 microbody, GO:0 005777 peroxisome, GO:00 19842 vitamin binding, GO:00 16614 oxidoreductase activity, acting on CH‐OH group of donors, GO:00 50660 flavin adenine dinucleotide binding, GO:00 16616 oxidoreductase activity, acting on the CH‐OH group of donors, NAD or NADP as acceptor, GO:00 51287 NAD binding) e) Additional GO enrichment analysis of apoptosis, oxidative stress, ATP metabolism, ROS response, stress‐activated MAPK cascades, and cell cycle DNA replication. f) GSEA enrichment plots of DEGs centralized in apoptosis, p53 signaling pathway, ferroptosis, and GSH metabolism. Heatmap representations of the DEGs in the g) apoptosis and h) ferroptosis in cells treated with SA‐Co@MoS_2_ + H_2_O_2_ + US (A1‐A3) compared to the control group (A4–A6).

To explore the underlying biological mechanisms of the SA‐Co@MoS_2_ nanoflowers‐mediated piezoelectric catalytic therapy in tumor treatment, a transcriptome analysis was conducted to assess alterations in messenger RNA (mRNA) profiles within HepG2 hepatocellular carcinoma cells post‐treatment. A comparison between the control and SA‐Co@MoS_2_ + H_2_O_2_ + US groups identifies 3631 differentially expressed genes (DEGs), with 1780 downregulated and 1851 upregulated (**Figure** **6**a). Principal Component Analysis (PCA) was employed to visualize the β‐diversity among tumor cells across different treatment groups (Figure 6b). Subsequent Kyoto Encyclopedia of Genes and Genomes (KEGG) analysis elucidates the biological functions and associated pathways of these altered mRNAs. DEGs following SA‐Co@MoS_2_ + H_2_O_2_ + US treatment are enriched in pathways such as the TNF signaling pathway, apoptosis, p53 signaling pathway, fatty acid metabolism, GSH metabolism, and ferroptosis (Figure 6c). To further investigate the biological processes underlying the antitumor effects induced by SA‐Co@MoS_2_ + H_2_O_2_ + US, Gene Ontology (GO) analysis was performed, revealing the alterations in cellular metabolism. This analysis highlights diverse biological processes, cellular components such as mitochondria and endoplasmic reticulum, and molecular functions including protein activity (Figure 6d). Additionally, GO enrichment analysis confirms the association of these DEGs with apoptosis, oxidative stress, ATP metabolism, ROS response, stress‐activated MAPK cascade, and cell cycle DNA replication, correlating with prior cellular experimental findings (Figure 6e). These results underscore the profound impact of the combined therapy on biological functions within cancer cells.

To gain deeper insights into the therapeutic mechanisms of this synergistic approach, Gene Set Enrichment Analysis (GSEA) was subsequently performed. The analysis demonstrates that the synergistic application of piezoelectricity and enzyodynamic therapy results in significantly positive enrichment scores for pathways associated with ferroptosis and apoptosis (Figure 6f). To visualize differential gene expression patterns, heatmap analyses were performed, highlighting the distinct differences in gene expression between the control and SA‐Co@MoS_2_ + H_2_O_2_ + US groups (Figure 6g,h). Compared to the control group, key genes associated with ferroptosis, including MAP1LC3B, TFRC, GSS, SLC39A14, and SLC40A1, as well as pivotal genes involved in apoptosis, such as TNF, MMP3, CASP3, GADD45A, TUBA1A, and ACTB, exhibit substantial expression changes in the SA‐Co@MoS_2_ + H_2_O_2_ + US group, suggesting that the synergistic therapy significantly modulates the processes of ferroptosis and apoptosis, underscoring its potential as an effective therapeutic strategy in cancer treatment.

### 2.7 In Vivo Piezoelectric and Enzyodynamic Synergistic Therapy

**Figure 7:**
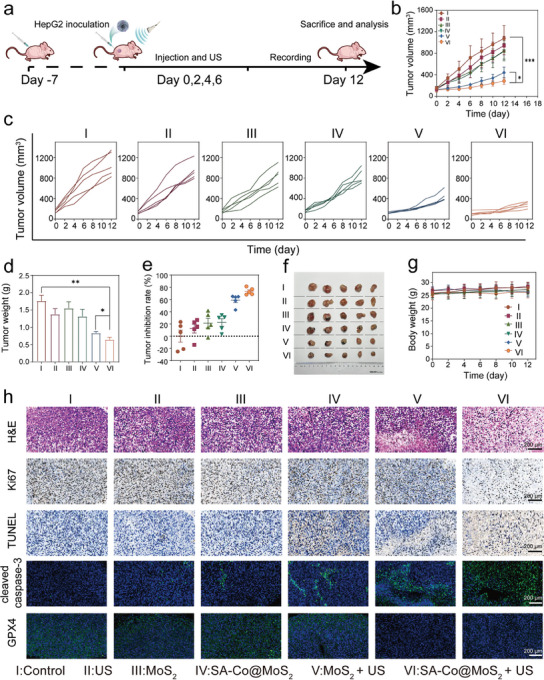
In vivo evaluation of synergistic therapy on HepG2 tumor‐bearing mice. a) Schematic representation of the treatment timeline for in vivo synergistic anticancer treatment (Created by Huiyantong Animation Technology Co., Ltd). b) Average tumor growth curves (*n* = 5) and c) Individual tumor growth curves for each mouse after different treatments. d) Average tumor weights measured post‐treatment for different treatments (*n* = 5). e) Tumor growth inhibition rates of mice after different treatments (*n* = 5). f) Photographs of tumors collected from different groups following 12 days of treatment (*n* = 5). g) Body weights of tumor‐bearing mice throughout the 12‐day treatment period (*n* = 5). h) Representative images of tumor slices in different treatment groups stained with H&E, Ki67, TUNEL, cleaved caspase‐3, and GPX4. Results are presented as means ± SD. Statistical significance was calculated using a one‐way ANOVA. *p < 0.05, **p < 0.01, ***p < 0.001.

Encouraged by the remarkable cellular‐level therapeutic results, we investigated the in vivo antitumor effects of SA‐Co@MoS_2_ nanoflowers mediated synergistic therapy. Initially, mouse models of liver tumors were established using HepG2 cells to evaluate the therapeutic effects (**Figure** **7**a), in which tumor‐bearing mice were randomly divided into six groups: I) Control; II) US; III) MoS_2_ nanoflowers; IV) SA‐Co@MoS_2_ nanoflowers; V) MoS_2_ nanoflowers + US; VI) SA‐Co@MoS_2_ nanoflowers + US. Compared to the control group, mice treated with MoS_2_ and SA‐Co@MoS_2_ nanoflowers exhibit mild tumor inhibition, with average volumes of 849 and 837 mm^3^, respectively, attributable to the enzyodynamic therapeutic effects of MoS_2_ and SA‐Co@MoS_2_ nanoflowers (Figure 7b,c). Combined US‐triggered piezoelectric catalysis significantly enhances tumor suppression in both groups, with average tumor volumes reducing to 442 mm^3^ for MoS_2_ nanoflowers + US group and 285 mm^3^ for SA‐Co@MoS_2_ nanoflowers + US group. The tumor growth inhibition rate for the SA‐Co@MoS_2_ + US group reaches 76.8% by day 12, surpassing the rates of other groups: 10.3% for US, 20.2% for MoS_2_ nanoflowers, 23.5% for SA‐Co@MoS_2_ nanoflowers, and 62.1% for MoS_2_ nanoflowers + US (Figure 7d–f). Importantly, bi‐daily monitoring of tumor volumes and body weights throughout the treatment period reveals no significant weight fluctuations across all groups, indicating the biological safety of the interventions (Figure 7g). To further assess the therapeutic efficacy, tumor sections underwent Hematoxylin and Eosin (H&E) staining, Terminal deoxynucleotidyl transferase dUTP nick end labeling (TUNEL) staining, Ki67 immunostaining, cleaved caspase‐3 and GPX4 immunofluorescence staining (Figure 7h). H&E, TUNEL, and cleaved caspase‐3 staining results indicate substantial tumor cell apoptosis in the MoS_2_ nanoflowers + US and SA‐Co@MoS_2_ nanoflowers + US groups compared to other groups. Immunohistochemical staining for Ki67 reveals marked inhibition of tumor proliferation following treatment with the SA‐Co@MoS_2_ nanoflowers + US. Furthermore, a significant reduction in GPX4 expression in the tumor tissues from the SA‐Co@MoS_2_ nanoflowers and SA‐Co@MoS_2_ nanoflowers + US groups underscores the potential of the treatment to induce ferroptosis.

**Figure 8:**
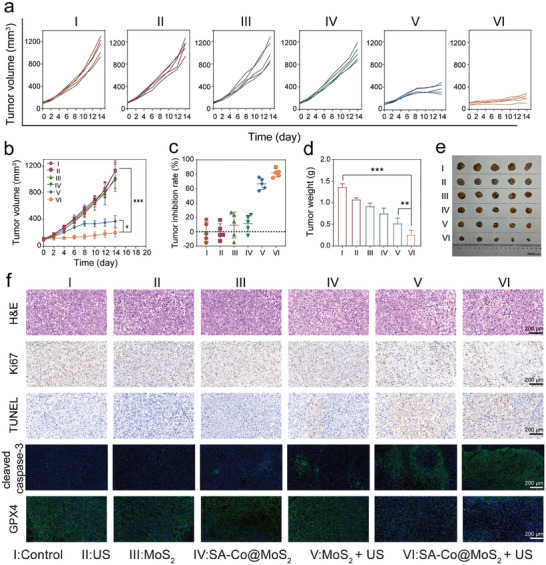
In vivo evaluation of synergistic therapy on 4T1 tumor‐bearing mice. a) Individual tumor growth curves and b) Average tumor growth curves for each mouse after different treatments (*n* = 5). c) Tumor growth inhibition rates of mice after different treatments (*n* = 5). d) Average tumor weights measured post‐treatment for different treatments (*n* = 5). e) Photographs of tumors collected from different groups following 14 days of treatment (*n* = 5). f) H&E, Ki67, TUNEL, cleaved caspase‐3, and GPX4 staining images of tumor slices from the tumor‐bearing mice in different treatment groups. Results are presented as means ± SD. Statistical significance was calculated using a one‐way ANOVA. *p < 0.05, **p < 0.01, ***p < 0.001.

To further assess the broad therapeutic nature of SA‐Co@MoS_2_ nanoflowers mediated piezocatalytic combined enzyodynamic therapy, an additional antitumor investigation was conducted on a 4T1 breast cancer model. The SA‐Co@MoS_2_ nanoflowers + US group demonstrates a remarkable tumor growth inhibition rate of 82.2%, significantly surpassing all other treatment groups (**Figure** **8**a–e). Importantly, throughout the experimental period, there are no notable reductions in body weight or significant toxic effects on vital organs (Figures S25–S27, Supporting Information), indicating a favorable safety profile for this therapeutic approach. Complementary histological analyses, including H&E staining, TUNEL staining, Ki‐67 immunostaining, and immunofluorescence staining for cleaved caspase‐3 and GPX4, further substantiate these findings. Tumor tissues from the SA‐Co@MoS_2_ nanoflowers + US group display the highest levels of apoptosis and ferroptosis, coupled with the lowest proliferation rates (Figure 8f), thereby providing compelling evidence for the robust antitumor efficacy of the treatment strategy.

## 3 Conclusion

In this study, we developed a strategy utilizing single‐atom Co doping to significantly enhance the piezocatalytic and enzyodynamic activities of MoS_2_ nanoflowers, culminating in a synergistic therapeutic effect against tumors (liver and breast tumors). The precise introduction of Co single atoms induces out‐of‐plane polarization, which not only generates abundant S vacancies but also narrows the intrinsic bandgap of MoS_2_ nanoflowers, thereby facilitating enhanced carrier separation and migration. This approach amplifies the material's capacity to mimic various biological enzymes, including ROS‐related POD‐mimicking and hypoxia‐reversing CAT‐mimicking activities, while significantly accelerating GSH consumption. The ROS generated through enhanced piezocatalytic properties and enzyodynamic activities can induce the expression of the pro‐apoptotic protein Bax and cleaved caspase‐3 while inhibiting the anti‐apoptotic protein Bcl‐2 and the ferroptosis‐related protein GPX4. Subsequent transcriptomic sequencing analysis reveals that DEGs are enriched in signaling pathways related to apoptosis, ferroptosis, oxidative stress, ATP metabolism, ROS response, and cell cycle DNA replication. In vitro cellular assays and in vivo animal studies demonstrate significant therapeutic outcomes associated with enhanced piezocatalytic and enzyodynamic therapy. Our findings not only establish a novel framework for developing high‐performance piezoelectric catalysts through polarization enhancement but also provide a robust theoretical and experimental foundation for employing single‐atom modification techniques to enhance the properties of piezoelectric biomaterials, thereby expanding their potential applications in tumor therapy.

## 4 Experimental Section

### Chemicals

Molybdenum trioxide (MoO_3_), thioacetamide (TAA), cobalt nitrate hexahydrate (Co(NO_3_)_2_·6H_2_O), N, N‐dimethylformamide (DMF), and urea were supplied by Sigma‐Aldrich. 1,3‐Diphenylisobenzofuran (DPBF), 5,5′‐dithiobis‐2‐nitrobenzoic acid (DTNB), 5,5‐dimethyl‐1‐pyrroline‐N‐oxide (DMPO), triacetonamine hydrochloride (TEMP), and 3,3′,5,5′‐tetramethyl‐benzidine (TMB) were purchased from Aladdin Chemical Reagent Co., Ltd. 2,7‐dichlorofluorescein diacetate (DCFH‐DA), JC‐1 staining kit, Calcein‐AM, propidium iodide (PI), and annexin V‐FITC/PI apoptosis detection kit were purchased from Beyotime Biotechnology. All chemicals were used as received without purification.

### Synthesis of Pristine MoS_2_ and SA‐Co@MoS_2_ Nanoflowers

MoO_3_ (30 mg), TAA (45 mg), urea (53 mg), and Co(NO_3_)_2_·6H_2_O (10 mg) were dissolved in 30 mL of DMF and then sonicated for 1 h. The mixture was transferred to an autoclave and heated at 200 °C for 20 h. After cooling naturally to room temperature, the product was collected by centrifugation at 13 000 rpm for 15 min and washed several times with ethanol and deionized water to obtain SA‐Co@MoS_2_ nanoflowers, which were vacuum dried at 60 °C overnight. The same method was also used to prepare pristine MoS_2_ nanoflowers, but without using Co(NO_3_)_2_·6H_2_O precursor in the solvothermal synthesis.

### Characterization of MoS_2_ and SA‐Co@MoS_2_ Nanoflowers

The size and surface charge of MoS_2_ and SA‐Co@MoS_2_ nanoflowers were analyzed by dynamic light scattering (DLS, Zetasizer Nano ZSE, Malvern Instrument Ltd., UK). Transmission electron microscope (TEM), energy‐dispersive X‐ray spectroscopy (EDS), and corresponding elemental analysis were recorded using a field emission transmission electron microscope (JEOL Company Ltd., Japan). X‐ray diffraction (XRD) patterns were recorded using an 18KW D/MAX2500V+/PC XRD instrument (Rigaku Co. Ltd., Tokyo, Japan). X‐ray photoelectron spectroscopy (XPS) was obtained on the Nexsa G2 from Thermo Fisher Scientific (Thermo Fisher Scientific Co. Ltd., USA). Aqueous ultraviolet‐visible‐near‐infrared (UV‐vis‐NIR) absorption spectra were determined on a UV‐3600 Plus Shimadzu spectrometer (Shimadzu Company Ltd., Japan). The absorbance of the solution at different wavelengths was recorded by the Varioskan LUX multi‐function enzyme labeling instrument (Thermo Fisher Scientific Co. Ltd., USA). Electron spin resonance (ESR) spectra were acquired on the EMXplus (Bruker Company Ltd., Germany). The quantitative detection results of Co, Mo, and S elements were obtained by inductively coupled plasma‐optical emission spectrometry (ICP‐OES) (Optima 7300 DV, PerkinElmer, USA).

### Electrochemical Tests

Electrochemical tests were conducted on an electrochemical workstation (CHI660D) in a 0.5 m Na_2_SO_4_ solution. The working electrode (sample‐coated ITO glass), counter electrode (Pt wire), and reference electrode (Ag/AgCl standard electrode) were used for the experiments. The working electrode was fabricated by depositing 100 µL of the suspension prepared from 10 mg of MoS_2_ and SA‐Co@MoS_2_ nanoflowers mixed with 50 µL of Nafion solution in ethanol onto ITO glass.

### GSH Depletion and Enzyme‐Mimicking Activities of MoS_2_ and SA‐Co@MoS_2_ Nanoflowers

GSH depletion by MoS_2_ and SA‐Co@MoS_2_ nanoflowers was determined by DTNB assay. MoS_2_ and SA‐Co@MoS_2_ nanoflowers (100 µg mL^−1^) were dispersed in the GSH solution (100 µm). Subsequently, 100 µL of the mixed solution was taken at different time points (0, 5, 10, 15, and 20 min), and the absorption peak intensity at 412 nm was recorded using a microplate reader after adding 5 µL of 1 mM DTNB. Additionally, 2 mL of the mixed solution was taken at different time points (0, 20, 40, 60, 80, 100, and 120 min), and the absorption at 412 nm was detected using UV‐vis absorption spectra after adding 20 µL of 1 mm DTNB. POD‐mimicking enzyme activity was assessed at room temperature using TMB as substrate. The absorbance of oxTMB was recorded at 652 nm using a microplate reader or a UV‐vis spectrophotometer. Specifically, 50 µL of MoS_2_ and SA‐Co@MoS_2_ nanoflowers solution (1 mg mL^−1^), 30 µL of TMB solution (1 mg mL^−1^), and 40 µL of aqueous H_2_O_2_ solution (100 mm) were sequentially added to buffers of different pHs (3.4, 4.5, 5.5, 6.0, and 7.4) to a final volume of 2 mL. Referring to the above method, different concentrations of H_2_O_2_ (0, 20, 40, 60, 80, and 100 mm) were added to the PBS solution at pH 4.5 and the kinetics were determined. The oxidation of TMB was measured using UV‐vis absorption spectra recording. A portable dissolved oxygen meter detected the CAT‐mimicking activities of MoS_2_ and SA‐Co@MoS_2_ nanoflowers. Under stirring conditions, 200 µL of H_2_O_2_ solution at different concentrations (2, 4, 6, and 8 µm) were added to 20 mL of aqueous solution of MoS_2_ and SA‐Co@MoS_2_ nanoflowers (50 mg mL^−1^). The dissolved oxygen content was recorded every 10 seconds during stirring. The Michaelis‐Menten constant was calculated by the Lineweaver‐Burk Plot: 1/V  =  Km/ Vmax (1/[S] + 1/Km).

### Evaluation of Piezoelectric Performance

30 µL of DPBF (1 mg mL^−1^) was added sequentially to 2 mL of water, along with 50 µL of MoS_2_ and SA‐Co@MoS_2_ nanoflowers solution (1 mg mL^−1^), and then sonicated for 5 min (1.0 MHz, 50% duty cycle, 1.0 W cm^−2^). During sonication, UV‐vis absorption spectra were measured every minute to determine the generation of ^1^O_2_. Using TEMP as the trapping agent, ESR spectra were used to measure the ROS generation efficiency of MoS_2_ and SA‐Co@MoS_2_ nanoflowers. 20 µL of TEMP was added to MoS_2_ and SA‐Co@MoS_2_ nanoflowers, followed by sonication for 5 min. The mixture was then transferred to a quartz tube to measure the ^1^O_2_ generation efficiency of MoS_2_ and SA‐Co@MoS_2_ nanoflowers.

### Cell Culture

HepG2 cells were purchased from the Cell Bank of the Chinese Academy of Sciences. HepG2 cells were cultured in a cell culture incubator at 37 °C with 5% CO_2_. The medium was a high glucose medium (DMEM) containing 10% fetal bovine serum (FBS), along with penicillin and streptomycin at a concentration of 100 µg mL^−1^.

### In Vitro Cellular Uptake

HepG2 cells were seeded in 12‐well plates and incubated with FITC‐labeled SA‐Co@MoS_2_ nanoflowers at a concentration of 50 µg mL^−1^ for 0, 2, 4, 6, 8, and 10 h. After incubation, the cells were washed three times with PBS. Subsequently, flow cytometry was employed to quantitatively analyze the fluorescence signal intensity of the cellular uptake of the SA‐Co@MoS_2_ nanoflowers.

### In Vitro Cytotoxicity Assay

The cytotoxicity of MoS_2_ and SA‐Co@MoS_2_ nanoflowers on HepG2 cells was evaluated. HepG2 cells were inoculated in 96‐well plates and allowed to adhere overnight. Afterwards, different concentrations of the materials (0, 25, 50, and 100 µg mL^−1^) were added to incubate with the cells in DMEM solution at 37 °C, pH 7.4, with 5% CO_2_ for 24 h. Cell viability was then tested using the CCK‐8 assay.

Additionally, different methods were used to investigate the effect of sonication on cell viability. HepG2 cells were incubated with different treatment groups (Control, US, MoS_2_, MoS_2_ + H_2_O_2_, MoS_2_ + US, MoS_2_ + H_2_O_2_ + US, SA‐Co@MoS_2_, SA‐Co@MoS_2_ + H_2_O_2_, SA‐Co@MoS_2_ + US, SA‐Co@MoS_2_ + H_2_O_2_ + US) for 8 h. The cells were then sonicated for 5 min (1.0 MHz, 50% duty cycle, 1.0 W cm^−2^), after which they were replaced with fresh complete medium and incubated for another 12 h. Finally, the cytotoxicity was examined using the CCK‐8 assay.

### Intracellular ROS Measurements

HepG2 cells were inoculated in confocal dishes for 12 h. Subsequently, they were incubated with different treatment groups (Control, US, MoS_2_, MoS_2_ + H_2_O_2_, MoS_2_ + US, MoS_2_ + H_2_O_2_ + US, SA‐Co@MoS_2_, SA‐Co@MoS_2_ + H_2_O_2_, SA‐Co@MoS_2_ + US, SA‐Co@MoS_2_ + H_2_O_2_ + US) for 8 h, followed by staining with DCFH‐DA for 30 min. The cells were then sonicated for 5 min (1.0 MHz, 50% duty cycle, 1.0 W cm^−2^), and the cells were gently washed three times with PBS. Finally, the cells were detected by laser confocal microscopy.

### Live/Dead Cell Staining

HepG2 cells were cultured and exposed to different treatment groups (Control, US, MoS_2_, MoS_2_ + H_2_O_2_, MoS_2_ + US, MoS_2_ + H_2_O_2_ + US, SA‐Co@MoS_2_, SA‐Co@MoS_2_ + H_2_O_2_, SA‐Co@MoS_2_ + US, SA‐Co@MoS_2_ + H_2_O_2_ + US) for 8 h. The cells were then sonicated for 5 min (1.0 MHz, 50% duty cycle, 1.0 W cm^−2^). After staining with Calcein AM and PI according to the manufacturer's protocol, the cells were imaged by laser confocal microscopy.

### Apoptosis Detection Assay

For a precise quantitative assessment of apoptosis‐induced cell death, HepG2 cells were initially seeded in 12‐well plates and allowed to adhere overnight. Subsequently, after specific treatment protocols, the cells underwent trypsinization and thorough washing procedures. The cells were next sonicated for 5 min (1.0 MHz, 50% duty cycle, 1.0 W cm^−2^). They were stained with annexin V‐FITC and PI, strictly adhering to the manufacturer's guidelines. This staining enabled the quantification of apoptotic cells through flow cytometry analysis.

### Assessment of Mitochondrial Membrane Potential and Intracellular ATP Levels

HepG2 cells were planted in 12‐well plates and allowed to adhere overnight and treated with different groups (Control, US, MoS_2_, MoS_2_ + H_2_O_2_, MoS_2_ + US, MoS_2_ + H_2_O_2_ + US, SA‐Co@MoS_2_, SA‐Co@MoS_2_ + H_2_O_2_, SA‐Co@MoS_2_ + US, SA‐Co@MoS_2_ + H_2_O_2_ + US) for 8 h. The cells were next sonicated for 5 min (1.0 MHz, 50% duty cycle, 1.0 W cm^−2^). The culture medium was replaced with a JC‐1 staining solution according to the manufacturer's protocol. Afterward, the cells were washed with PBS three times before flow cytometry analysis.

The intracellular level of ATP was detected by ATP assay kits. Similar to mitochondrial membrane potential assay detection, HepG2 cells were irradiated with US (1.0 MHz, 50% duty cycle, 1.0 W cm^−2^, 5 min). After 12 h, HepG2 cells were lysed and the supernatant was immediately collected by centrifugation for detection of ATP level in vitro using assay kits.

### Cell cycle and Apoptosis Analysis

HepG2 cells were planted in 12‐well plates and allowed to adhere overnight before being treated with different groups (Control, US, MoS_2_, MoS_2_ + H_2_O_2_, MoS_2_ + US, MoS_2_ + H_2_O_2_ + US, SA‐Co@MoS_2_, SA‐Co@MoS_2_ + H_2_O_2_, SA‐Co@MoS_2_ + US, SA‐Co@MoS_2_ + H_2_O_2_ + US) for 8 h. The cells were next sonicated for 5 min (1.0 MHz, 50% duty cycle, 1.0 W cm^−2^). After treatment, the cells were harvested and washed with ice‐cold PBS three times. For the cell cycle assay, the cells were resuspended with 1 mL of 75% cold ethanol and stored at −20 °C overnight. Then, the cells were centrifuged and washed with ice‐cold PBS three times and incubated with 500 µL of RNase (0.1 mg mL^−1^) and PI (50 µg mL^−1^) at 4 °C for 30 min. All data were collected by flow cytometry and analyzed using Flowjo software.

### Assessment of Intracellular GSH Levels

HepG2 cells were seeded onto 6‐well plates and incubated for 12 h. Subsequently, the cells were co‐incubated with different concentrations of MoS_2_ and SA‐Co@MoS_2_ nanoflowers (0, 12.5, 25, and 50 µg mL^−1^) or according to different treatment groups for 24 h. Following the manufacturer's instructions, a GSH assay kit was employed to measure GSH content. The absorbance at 412 nm was measured using a microplate reader.

### Western Blot Analysis

HepG2 cells were seeded onto 6‐well plates and incubated for 12 h before being treated with different groups (Control, SA‐Co@MoS_2_ + H_2_O_2_ + US) for 8 h. Cell lysates were collected and subjected to electrophoresis in denaturing polyacrylamide gels for analysis.

### Biosafety Evaluation

Healthy Kunming mice (*n* = 3) were intravenously administered MoS_2_ and SA‐Co@MoS_2_ nanoflowers (20 mg kg^−1^) and euthanized on the 10th day after injection. Untreated healthy mice (*n* = 3) served as the control group. Before euthanasia, blood samples were collected from the mice for a comprehensive blood panel and blood biochemistry assessments. Following euthanasia, mice from each group were dissected, and their primary organs (heart, liver, spleen, lung, and kidney) were excised for histological analysis via H&E staining.

### In Vivo Tumor Suppression Experiments

To develop a tumor model, HepG2 and 4T1 cells (5 × 10^6^) suspended in 100 µL PBS were subcutaneously injected into the right leg of each Balb/c mouse. When the tumor volume reached ≈ 100 mm^3^, mice were randomly divided into six groups consisting of five mice in each group: I) Control, II) US, III) MoS_2_ nanoflowers, IV) SA‐Co@MoS_2_ nanoflowers, V) MoS_2_ nanoflowers + US, and VI) SA‐Co@MoS_2_ nanoflowers + US. Then, 100 µL of MoS_2_ and SA‐Co@MoS_2_ nanoflowers (20 mg kg^−1^) were injected intratumorally into the mice respectively. In the US irradiation groups, tumors were treated with ultrasound irradiation (1.0 MHz, 50% duty cycle, 1 W cm^−2^, 5 min) on days 0, 2, 4, and 6, following an intratumoral injection 1 h prior to the therapy session. During the treatments, the tumor volume and body weight of the tumor‐bearing mice were recorded every two days. The tumor volume was calculated by the following formula: volume  =  length  × width2/2. Finally, the mice were euthanized, and the tumors were resected for histological staining, including H&E, Ki67, TUNEL, cleaved caspase‐3, and GPX4, to examine the anticancer effect.

### Computational Simulation Section

The system energy calculations were performed by using the Vienna ab initio simulation package (VASP) code.^[^ ^40^ ^]^ The projector augmented wave (PAW) method was used to describe the ionic potential and the Perdew‐Burke‐Ernzerhof (PBE) functional was used to describe the exchange‐correlation interactions.^[^ ^41^ ^]^ The plane‐wave kinetic energy cutoff was 500 eV. All the calculations used a 3×3×1 Monkhorst‐Pack k‐point mesh for the Brillouin zone sampling.^[^ ^42^ ^]^ For structure relaxation, the energy convergence criterion for electronic relaxation is 1 × 10^−5^ eV and the ionic relaxation was performed until all forces were smaller than 0.01 eV/Å. The free energy(ΔG) of each reduction step was obtained at zero bias potential using,^[^ ^43^ ^]^

(4) $$\Delta G=\Delta E+\Delta E_{\mathrm{ZPE}}+T\Delta S$$

where ΔE was the reaction energy, ΔE_ZPE_ was the difference in zero‐point energies, *T* was the temperature (298K) and ΔS is the reaction entropy.

### Statistical Analysis

All data were analyzed by GraphPad Prism 8 (GraphPad Software). Data were expressed as the mean ± standard deviation (SD), and each data quantitative evaluation was repeated at least three times. The statistical significance of the data was assessed using one‐way analysis of variance (ANOVA) tests. The significance level in the statistical analyses was defined as *p < 0.05, **p < 0.01, and ***p < 0.001.

## Conflict of Interest

The authors declare no conflict of interest.

## Supporting information



Supporting Information

## Data Availability

The data that support the findings of this study are available from the corresponding author upon reasonable request.
