# Supplementary material for: Cobalt Single‐Atom Intercalation in Molybdenum Disulfide Enhances Piezocatalytic and Enzyodynamic Activities for Advanced Cancer Therapeutics
Source: Adv Sci (Weinh). 2025 Feb 14;12(14):2415485. doi: 10.1002/advs.202415485 (PMC11984846; doi:10.1002/advs.202415485)
Supplement: Supplementary file 1 — Supporting Information [file ADVS-12-2415485-s001.docx]

**Supporting Information**

**Cobalt Single-Atom Intercalation in Molybdenum Disulfide Enhances** **Piezocatalytic and Enzyodynamic Activities for Advanced Cancer Therapeutics**

*Haomiao Bai, Sujun Ding, Yanfei Dai, Jiefu Liu, Huangjing Chen, Wei Feng*, Dehong Yu*, Yu Chen*, and Xuejun Ni**

H. Bai, S. Ding, J. Liu, H. Chen, Prof. X. Ni

Department of Medical Ultrasound, Affiliated Hospital of Nantong University, Nantong 226001, P.R. China.

Email: dyfnxj213@163.com (Prof. X. Ni).

Prof. W. Feng, A/Prof. D. Yu, Prof. Y. Chen

Materdicine Lab, School of Life Sciences, Shanghai University, Shanghai 200444, P. R. China.

Email: fengw@shu.edu.cn (Prof. W. Feng); dehongyu@shu.edu.cn (A/Prof. D. Yu); chenyuedu@shu.edu.cn (Prof. Y. Chen).

Dr. Y. Dai

Radiology Department, Branch of Affiliated Hospital of Nantong University, Nantong 226001, P. R. China.

Prof. Y. Chen

Shanghai Institute of Materdicine, Shanghai 200051, P. R. China.

**Supplementary Figures**


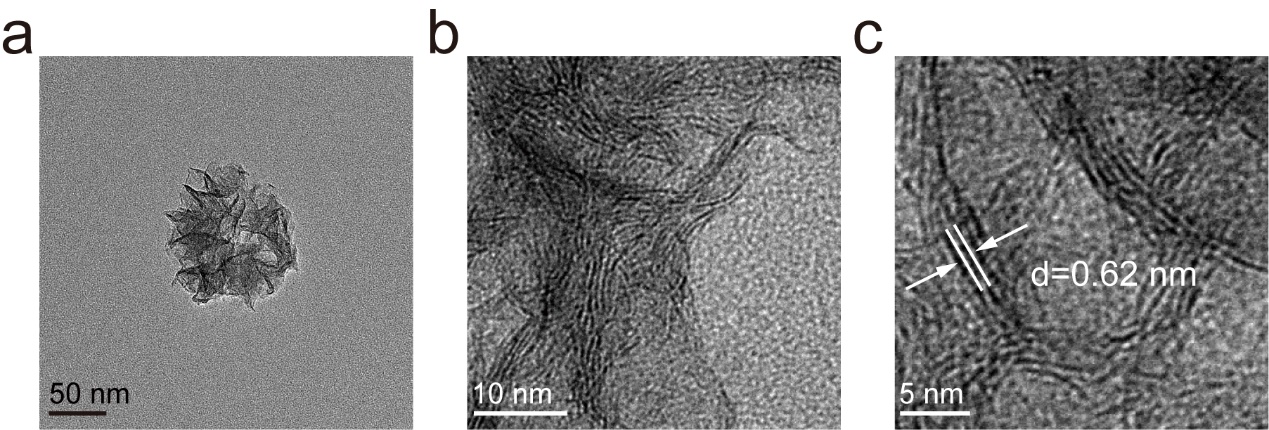


**Figure S1.** (a, b) TEM images of MoS_2_ nanoflowers. (c) HRTEM image of MoS_2_ nanoflowers.


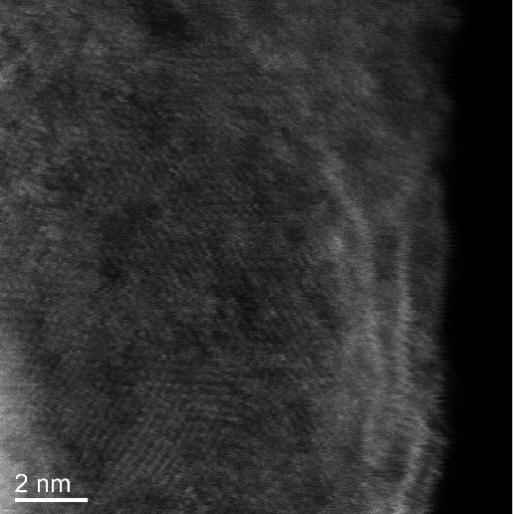


**Figure S2.** Spherical aberration-corrected TEM image of SA-Co@MoS_2_ nanoflowers.


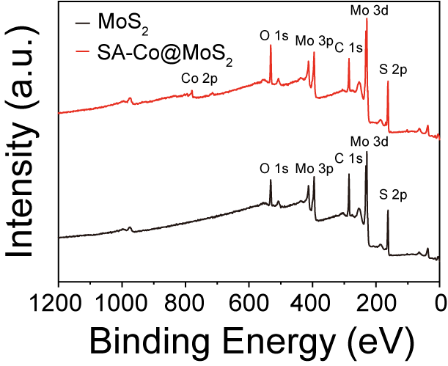


**Figure S3.** XPS spectra of SA-Co@MoS_2_ and MoS_2_ nanoflowers.


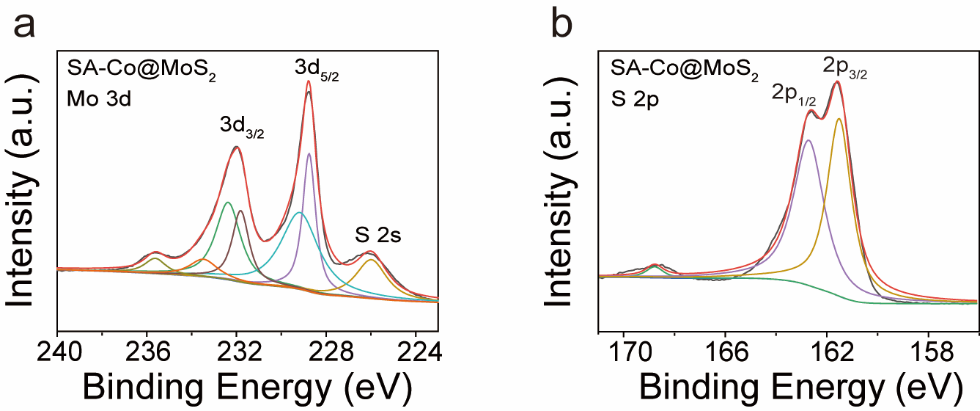


**Figure S4.** (a) Mo 3d and (b) S 2p XPS spectra of SA-Co@MoS_2_ nanoflowers.


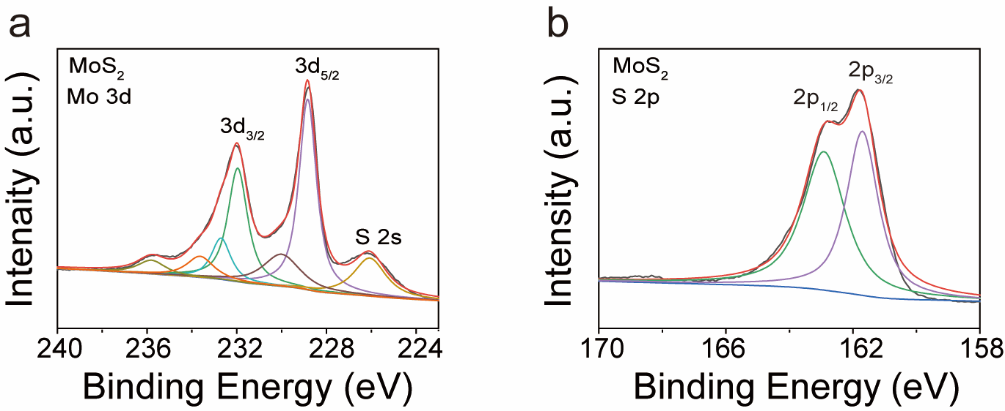


**Figure S5.** (a) Mo 3d and (b) S 2p XPS spectra of MoS_2_ nanoflowers.


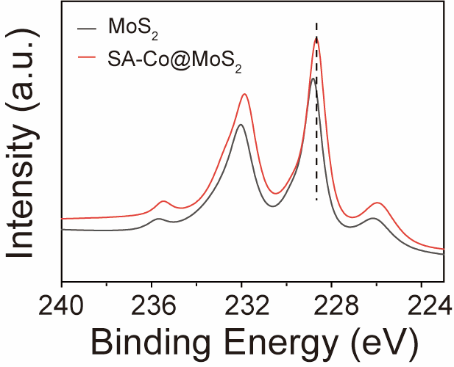


**Figure S6.** Mo 3d XPS spectra of SA-Co@MoS_2_ and MoS_2_ nanoflowers.


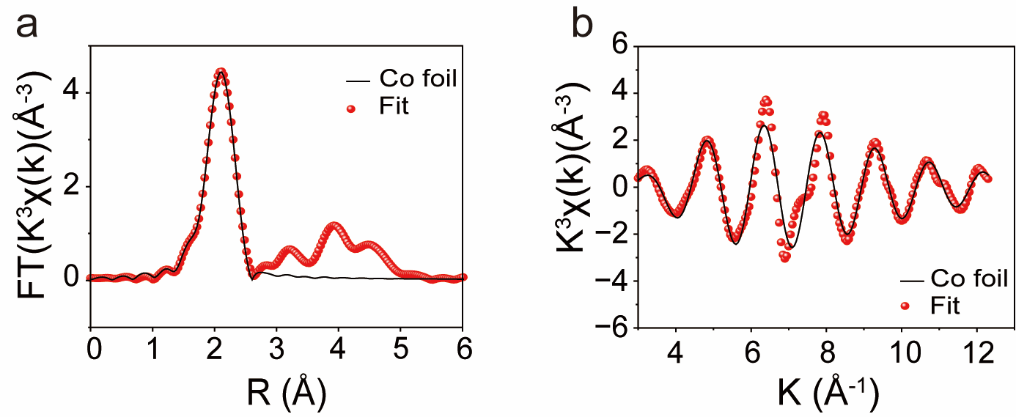


**Figure S7.** EXAFS fitting of Co foil in (a) R space and (b) k space.


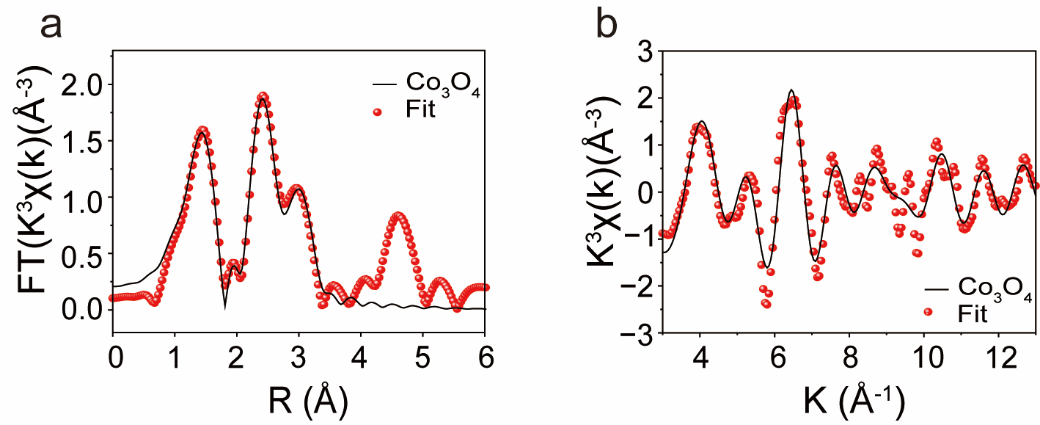


**Figure S8.** EXAFS fitting of Co_3_O_4_ in (a) R space and (b) k space.


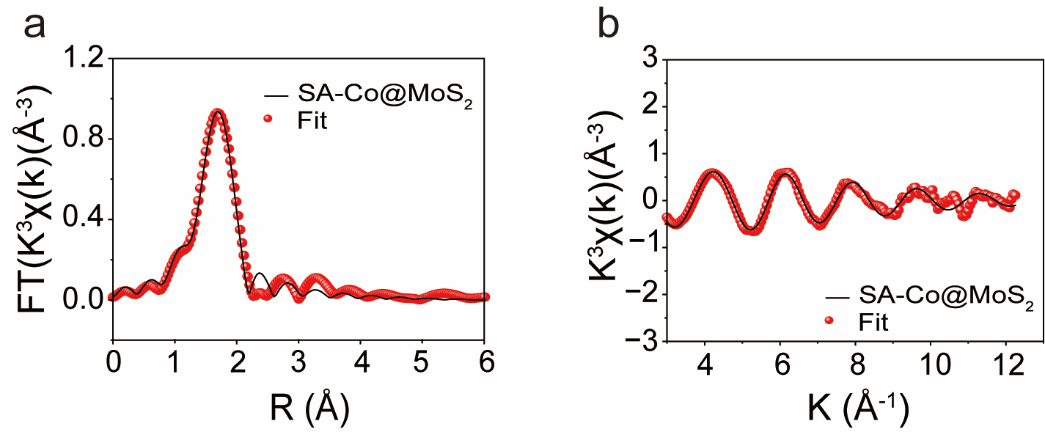


**Figure S9.** EXAFS fitting of SA-Co@MoS_2_ nanoflowers in (a) R space and (b) k space.


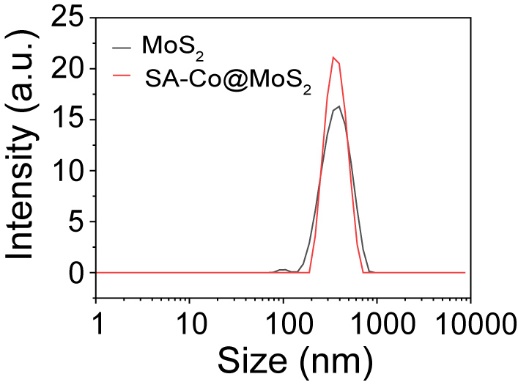


**Figure S10.** Hydrodynamic diameter of SA-Co@MoS_2_ and MoS_2_ nanoflowers dispersed in PBS.


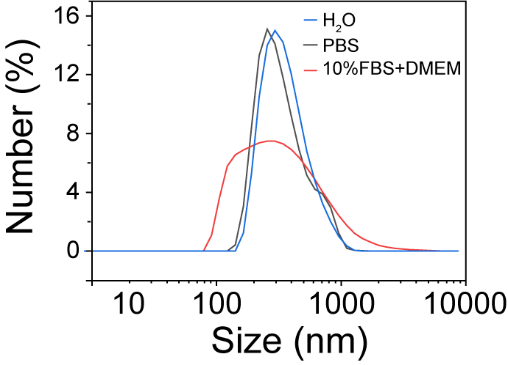


**Figure S11.** Hydrodynamic diameter of SA-Co@MoS_2_ nanoflowers in different physiological solutions.


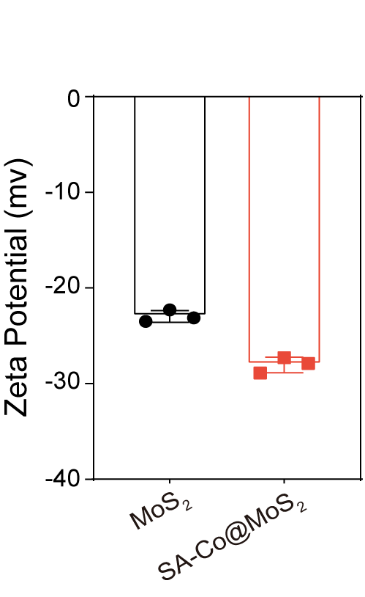


**Figure S12.** Zeta potentials of SA-Co@MoS_2_ and MoS_2_ nanoflowers dispersed in deionized water (n = 3).


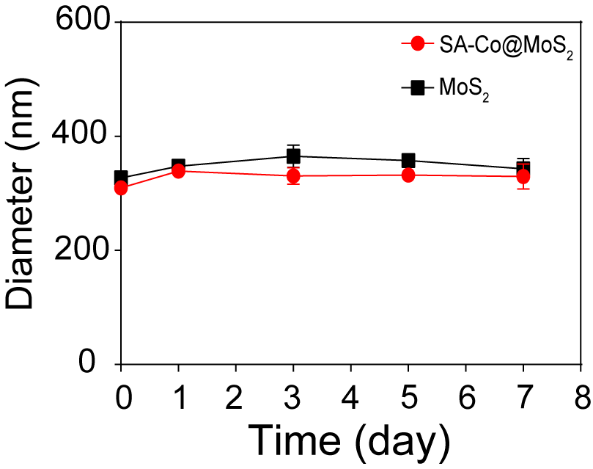


**Figure S13.** The stability of SA-Co@MoS_2_ and MoS_2_ nanoflowers dispersed in PBS solutions over time (n=3).


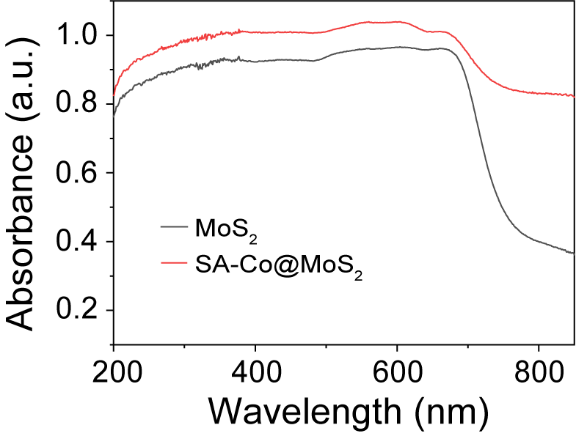


**Figure S14.** UV-Vis diffuse reflection spectra of MoS_2_ and SA-Co@MoS_2_ nanoflowers.


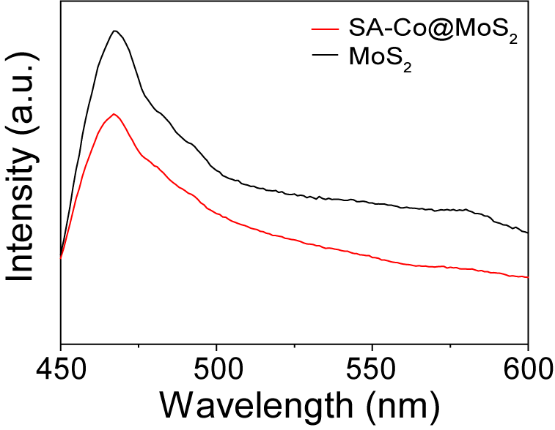


**Figure S15.** Photoluminescence spectra of MoS_2_ and SA-Co@MoS_2_ nanoflowers.


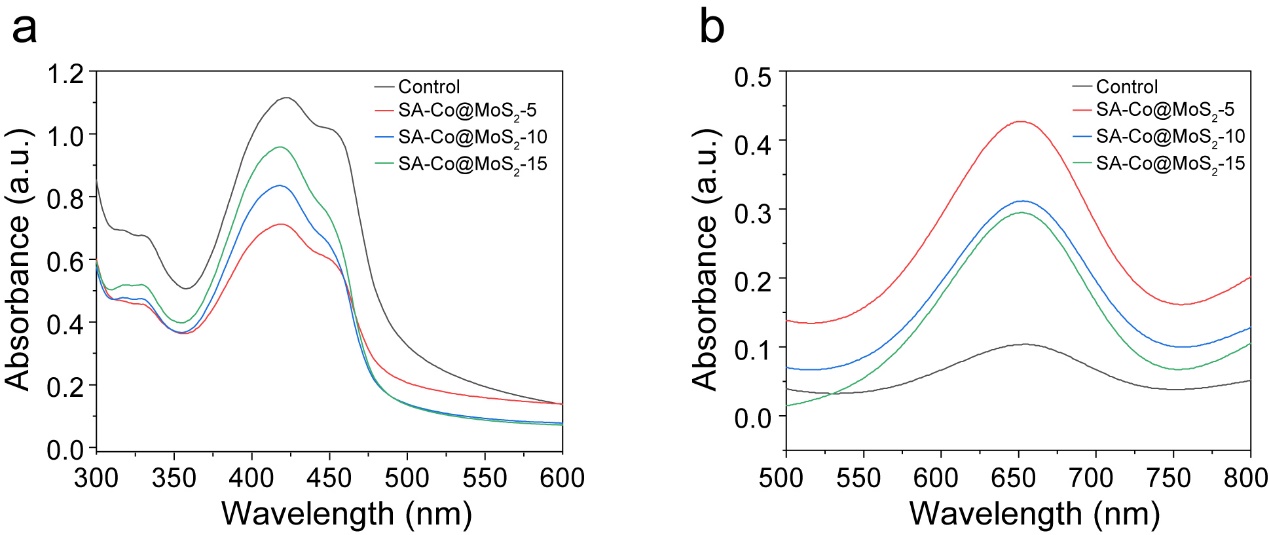


**Figure S16.** UV-Vis absorption spectra of (a) DPBF solutions incubated with SA-Co@MoS_2_-5, SA-Co@MoS_2_-10, and SA-Co@MoS_2_-15 under ultrasound irradiation (1.0 MHz, 1.0 W cm⁻², 50% duty cycle) for 5 minutes, and (b) SA-Co@MoS_2_-5, SA-Co@MoS_2_-10, and SA-Co@MoS_2_-15 incubated with H₂O₂ for 5 minutes.


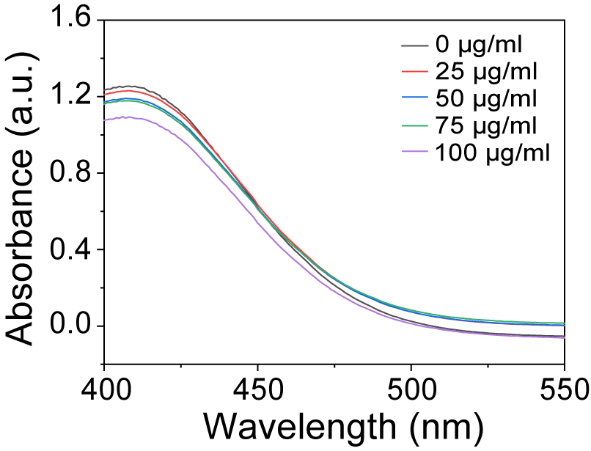


**Figure S17.** The GSH consumption of MoS_2_ nanoflowers using DTNB assay at different concentrations.


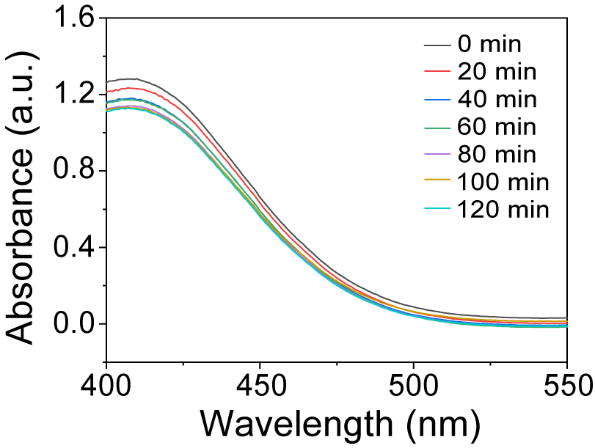


**Figure S18.** The GSH consumption of MoS_2_ nanoflowers using DTNB assay across different time points.


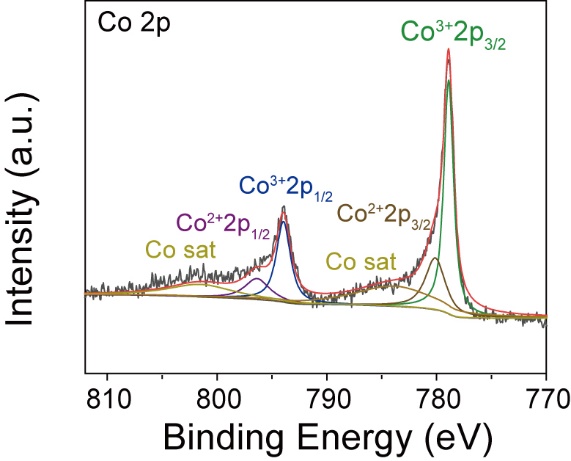


**Figure S19.** Co 2p XPS spectrum of SA-Co@MoS_2_ nanoflowers after reaction with GSH in ddH_2_O.


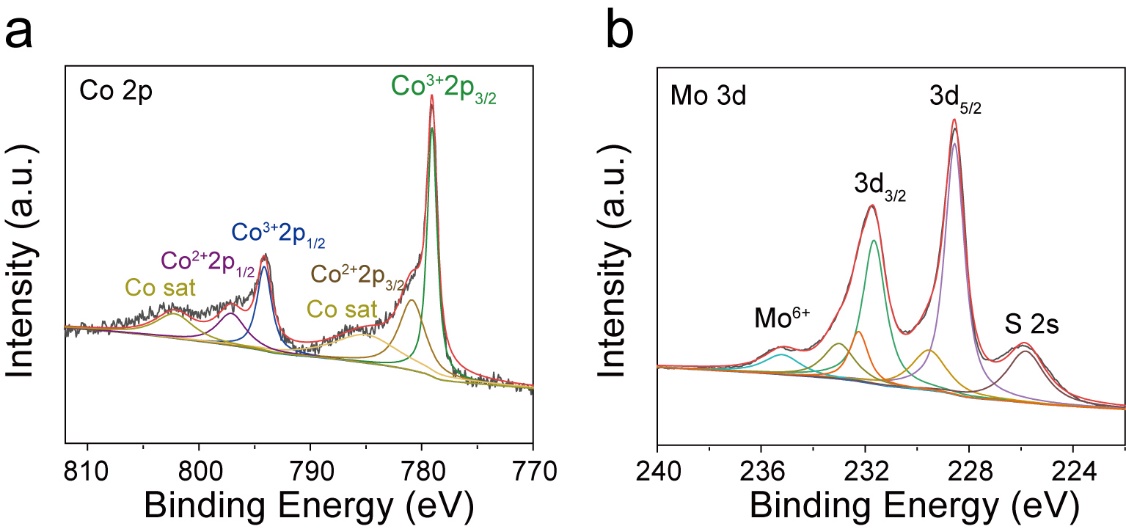


**Figure S20.** (a) Co 2p and (b) Mo 3d XPS spectra of SA-Co@MoS_2_ nanoflowers after reaction with H_2_O_2_ in ddH_2_O.


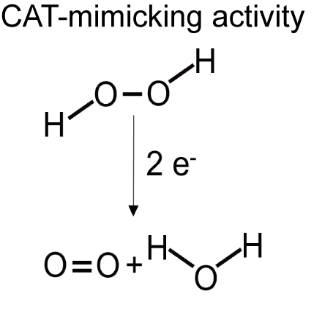


**Figure S21.** Schematic diagram of CAT-mimicking catalytic procedure of MoS_2_ and SA-Co@MoS_2_ nanoflowers.


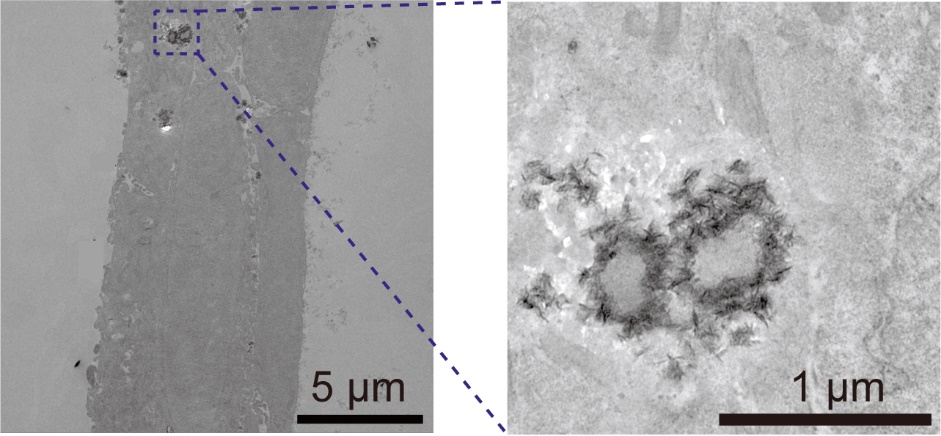


**Figure S22.** Bio-TEM images of HepG2 cancer cells after treatment with SA-Co@MoS_2_ nanoflowers.


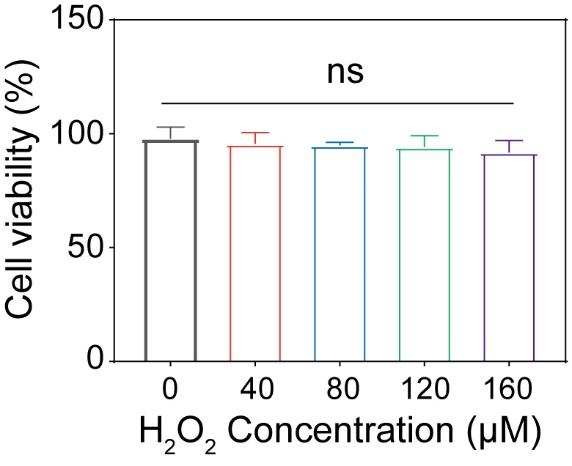


**Figure S23.** Relative cell viability of HepG2 cells after treatment with H_2_O_2_ at different concentrations for 24 hours (n=3).


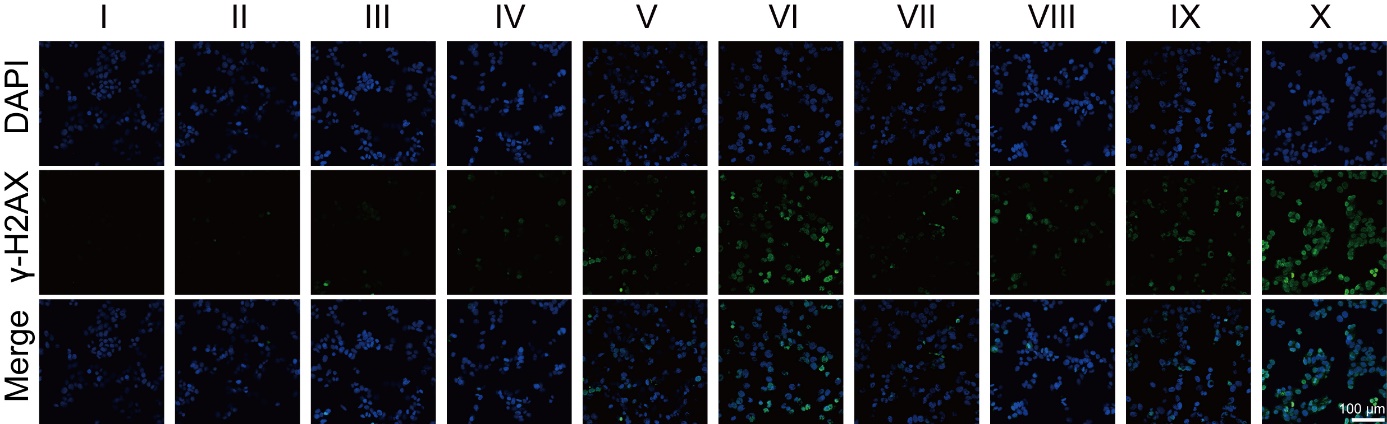


**Figure S24.** CLSM images of γ-H2AX-stained HepG2 cells after different treatments. I: Control; II: US; III: MoS_2_; IV: MoS_2_ + H_2_O_2_; V: MoS_2_ + US; VI: MoS_2_ + H_2_O_2_ + US; VII: SA-Co@MoS_2_; VIII: SA-Co@MoS_2_ + H_2_O_2_; IX: SA-Co@MoS_2_ + US; X: SA-Co@MoS_2_ + H_2_O_2_ + US.


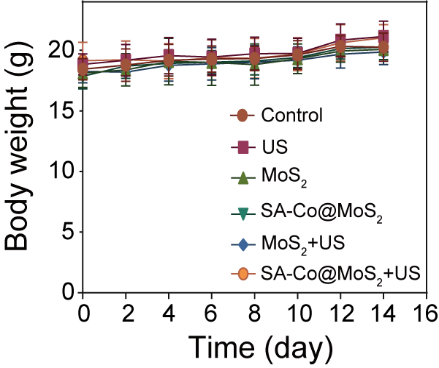


**Figure S25.** Body weights of 4T1 tumor-bearing mice during 14 days treatment (n=5).


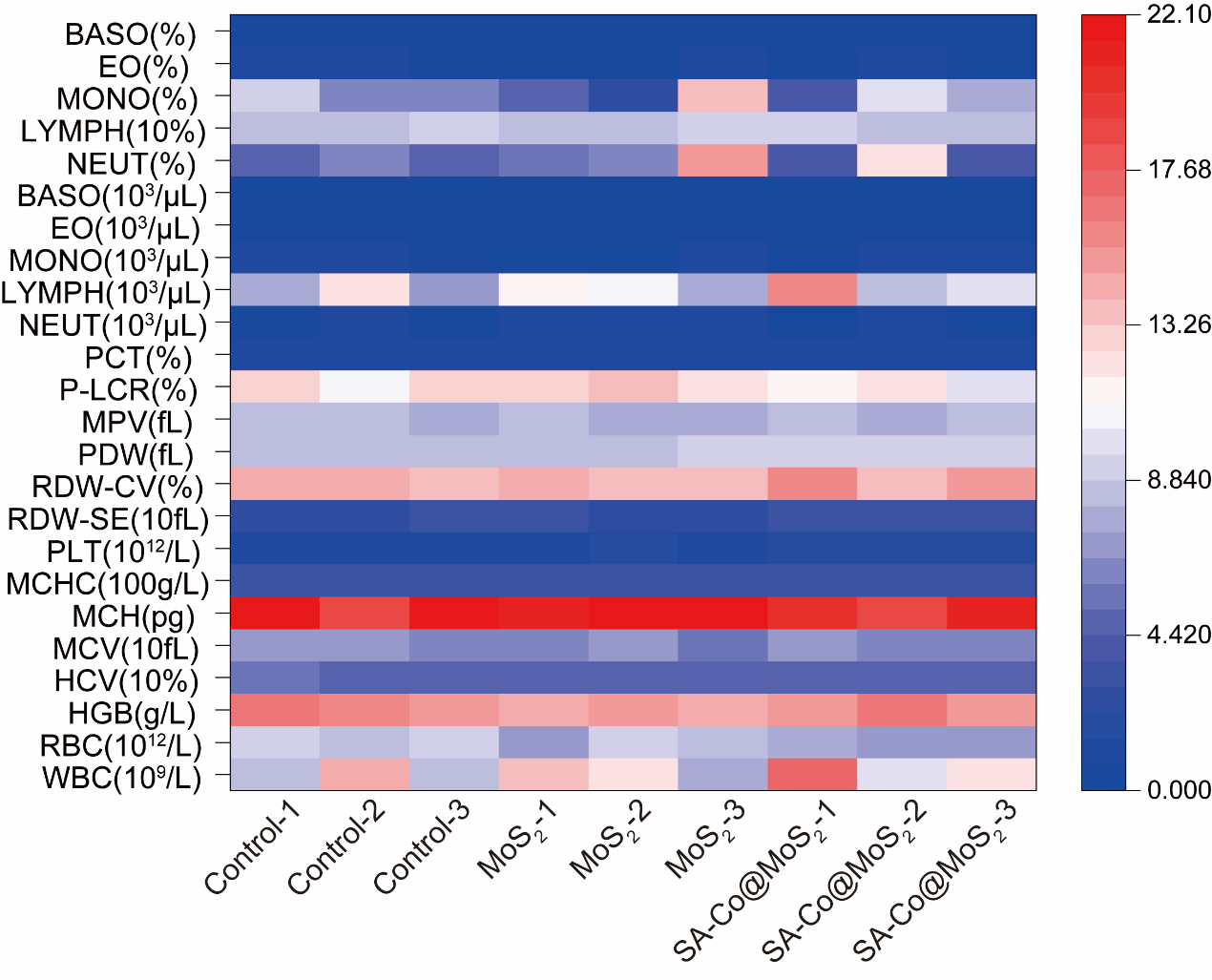


**Figure S26.** Hematological parameters of mice after different treatments.


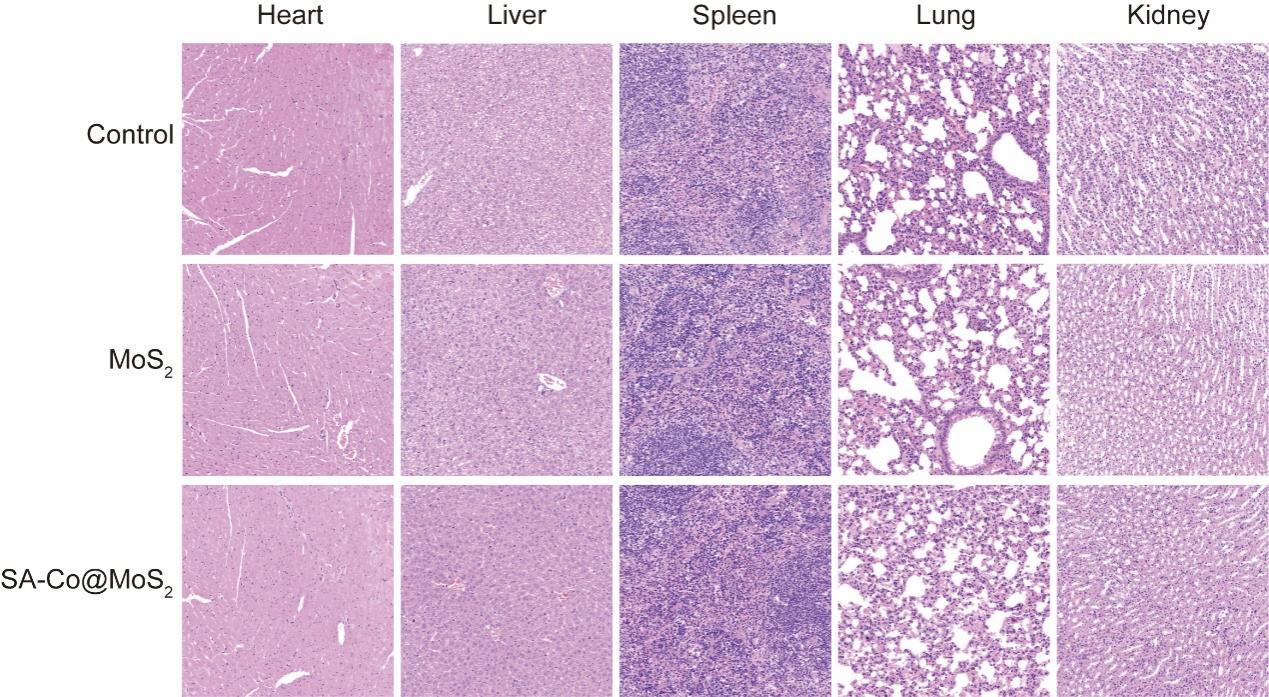


**Figure S27.** Histological sections collected from key organs, including the heart, liver, spleen, lungs, and kidneys, following a 10-day period of MoS_2_ and SA-Co@MoS_2_ administration via tail vein injection.

**Table S1.** EXAFS fitting parameters of different samples at the Co K-edge.

| Sample | Shell | *CN^a^* | *R*(Å)*^b^* | *σ*^2^(Å^2^)*^c^* | Δ*E*_0_(eV)*^d^* | *R* factor |
| --- | --- | --- | --- | --- | --- | --- |
| Co foil | Co-Co | 12* | 2.49 ± 0.1 | 0.0059 | -3.9 | 0.0032 |
| Co_3_O_4_ | Co-O1 | 2* | 1.91 ± 0.1 | 0.0015 | -5.4 | 0.0180 |
|  | Co-O2 | 4* | 1.93 ± 0.1 | 0.0080 | -5.4 |  |
|  | Co-Co1 | 6* | 2.87 ± 0.1 | 0.0053 | -7.1 |  |
|  | Co-Co2 | 6* | 3.37 ± 0.1 | 0.0039 | -7.1 |  |
| SA-Co@MoS_2_ | Co-S | 2.8 ± 0.3 | 2.21 ± 0.1 | 0.0050 | -8.0 | 0.0038 |

*^a^CN*, coordination number; *^b^R*, distance between absorber and backscatter atoms; *^c^σ*^2^, Debye-Waller factor to account for both thermal and structural disorders; *^d^ΔE*_0_, inner potential correction; *R* factor indicates the goodness of the fit. S_0_^2^ was fixed to 0.70. A reasonable range of EXAFS fitting parameters: 0.600 < *Ѕ*_0_^2^ < 1.000; *CN >* 0; *σ*^2^ > 0 Å^2^; |Δ*E*_0_| < 15 eV; *R* factor < 0.02.

**Table S2.** The parameters of time-resolved transient photoluminescence spectra.

|  | τ_1_ (ns) | τ_2_ (ns) | B1 | B2 | τ_ave_ (ns) |
| --- | --- | --- | --- | --- | --- |
| MoS_2_ | 1.96 | 1.16 | 1751.195 | 136.6542 | 2.66 |
| SA-Co@MoS_2_ | 1.70 | 1.04 | 1434.837 | 83.16071 | 2.18 |

**Table S3.** Bond lengths and angles of the geometries for Co on the Mo atop site, S substitution and the hollow site of MoS_2_ nanoflowers.

| Bond angle (°) | Mo-S | Co-S | Co-Mo |
| --- | --- | --- | --- |
| MoS_2_ | 80.62 | / | / |
| Mo top site | 81.09 | 98.41 | 83.62 |
| S substitution | 81.47 | 89.83 | 43.57 |
| Hollow site | 81.28 | 101.71 | 60.17 |

| Bond length (Å) | Mo-S | Co-S | Co-Mo |
| --- | --- | --- | --- |
| MoS_2_ | 2.40 |  |  |
| Mo top site | 2.46 | 2.03 | 2.59 |
| S substitution | 2.49 | 2.25 | 4.23 |
| Hollow site | 2.42 | 2.17 | 3.19 |
